# Not all errors are alike: modulation of error-related neural responses in musical joint action

**DOI:** 10.1093/scan/nsab019

**Published:** 2021-02-10

**Authors:** Anita Paas, Giacomo Novembre, Claudia Lappe, Peter E Keller

**Affiliations:** The MARCS Institute for Brain, Behaviour, and Development, Western Sydney University, Penrith, NSW 2751, Australia; Neuroscience of Perception and Action Lab & Neuroscience and Behaviour Lab, Italian Institute of Technology (IIT), Rome, 00161, Italy; Department of Medicine, Institute for Biomagnetism and Biosignalanalysis, University of Muenster, Muenster, 48149, Germany; The MARCS Institute for Brain, Behaviour, and Development, Western Sydney University, Penrith, NSW 2751, Australia

**Keywords:** performance monitoring, errors, joint action, electroencephalography (EEG), piano performance

## Abstract

During joint action, the sense of agency enables interaction partners to implement corrective and adaptive behaviour in response to performance errors. When agency becomes ambiguous (e.g. when action similarity encourages perceptual self–other overlap), confusion as to who produced what may disrupt this process. The current experiment investigated how ambiguity of agency affects behavioural and neural responses to errors in a joint action domain where self–other overlap is common: musical duos. Pairs of pianists performed piano pieces in synchrony, playing either the same pitches (ambiguous agency) or different pitches (unambiguous agency) while electroencephalography (EEG) was recorded for each individual. Behavioural and event-related potential results showed no effects of the agency manipulation but revealed differences in how distinct error types are processed. Self-produced ‘wrong note’ errors (substitutions) were left uncorrected, showed post-error slowing and elicited an error-related negativity (ERN) peaking before erroneous keystrokes (pre-ERN). In contrast, self-produced ‘extra note’ errors (additions) exhibited pre-error slowing, error and post-error speeding, were rapidly corrected and elicited the ERN. Other-produced errors evoked a feedback-related negativity but no behavioural effects. Overall findings shed light upon how the nervous system supports fluent interpersonal coordination in real-time joint action by employing distinct mechanisms to manage different types of errors.

## Introduction

Performance errors are informative, naturally occurring, unexpected events with consistent behavioural and neural responses that manifest across a variety of task domains. In sequential tasks such as piano performance, errors result in pre- and post-error slowing (Maidhof *et al.*, 2009; Ruiz *et al.*, 2009). Electroencephalographic (EEG) recordings show a consistent pattern of error-related neural activity reflected in event-related potentials (ERPs). The error-related negativity (ERN) evoked potential peaks around 50–100 ms post-error (for a review, see Gehring *et al.*, 2012). In sequential tasks, ERN latency shifts, peaking ∼50 ms before error onset, and is referred to as the pre-ERN instead of the ERN (Maidhof *et al.*, 2009, 2013; Ruiz *et al.*, 2009; Kalfaoğlu *et al.*, 2018). The ERN/pre-ERN is followed by the error positivity (Pe), peaking between 200 and 500 ms post-error (for a review, see Overbeek *et al.*, 2005; Gehring *et al.*, 2012). The Pe component is sometimes divided into an early, frontocentral Pe, peaking between 180 and 200 ms, and a late, parietal Pe, peaking around 300 ms (Van Veen and Carter, 2002; Ruchsow *et al.*, 2005; Ullsperger *et al.*, 2014b).

Another error-related ERP is the feedback-related negativity (FRN). The FRN peaks around 250 ms after feedback onset and is elicited upon error confirmation that is only verifiable through information about the action outcome, regardless of who performed the action (Miltner *et al.*, 1997; Gehring *et al.*, 2012). Thus, the perception of an error committed by another individual, which can occur during passive action observation or during joint action tasks, elicits the FRN (Nieuwenhuis *et al.*, 2004; Loehr *et al.*, 2015). Additionally, in cooperative task contexts, co-actors slow their behavioural responses after errors regardless of who committed them (De Bruijn *et al.*, 2012). Joint action scenarios requiring precise real-time interpersonal coordination, as in musical ensemble performance, present a complex case where the consequences of errors affect multiple individuals asymmetrically. Corrective measures may be required by the individual who committed the error, by the co-performers or by both, while all performers are typically expected to continue to act fluently in time with one another.

A theoretical model based on music ensemble performance posits that precise yet flexible interpersonal coordination is achieved through internal models that use representations of transformations between motor commands and sensory experiences to drive covert simulations of one’s own and other’s actions (Keller *et al.*, 2016). ‘Self’ internal models play a role in planning and prediction of one’s own actions (see Wolpert *et al.*, 1998), while ‘other’ internal models generate predictions about co-performers’ actions (Wolpert *et al.*, 2003). ‘Self’ plans and ‘other’ predictions are compared against shared goals and actual sensory feedback (including auditory, proprioceptive, visual and tactile feedback), and adjustments to the ‘self’ model can be made online to reduce discrepancies as joint performance progresses (Keller *et al.*, 2016; Harry and Keller, 2019). Importantly, this comparison process relies on a distinction between information related to the actions of self and other (cf. Pacherie, 2012; Kahl and Kopp, 2018).

With multiple actors, self–other distinction may become blurred when individuals produce actions that are similar due to perceptual overlap on temporal or spatial dimensions (Van Der Wel, 2015; Van Der Weiden *et al.*, 2019). Self–other blurring can lead to ambiguity in agency, that is, the experience of controlling one’s own actions and their external effects (Haggard and Tsakiris, 2009). Agency ambiguity is characterized by each individual feeling ownership of an action and its outcome (Farrer and Frith, 2002). Agency ambiguity can vary in degree. The ambiguity may be equal among co-performers if all have access to the same ambiguous information of the action outcome. However, some co-performers may have less ambiguous information about the action outcome, while others may have more ambiguous information (e.g. because performers may not be able to see and hear each other equally), and further confusion can arise through action similarity and feedback similarity. The challenge for the brain under conditions of agency ambiguity during joint action is to resolve the self–other distinction in order to maintain autonomous control of one’s own actions while generating accurate predictions for others’ actions (Keller *et al.*, 2016).

The current study investigated behavioural and neural responses to naturally occurring errors in a musical joint action task where agency was ambiguous or unambiguous. The specific aim was to test whether agency ambiguity affects responses to pitch errors committed by self versus other at levels of behavioural timing and underlying brain processes, indexed by ERPs. Thus, pairs of pianists played memorised right-handed piano pieces at different pitches (i.e. unambiguous condition) or the same pitch (i.e. ambiguous condition) as behavioural performance and EEG activity were recorded. Measures of keystroke timing, including interpersonal asynchronies (indexing coordination) and inter-keystroke intervals (IKIs) (reflecting tempo), were analysed to examine error-related behavioural responses for self-produced and other-produced errors relative to correct responses. Based on previous research identifying different types of error in piano performance (e.g. Palmer and Van De Sande, 1993), we examined note substitutions (wrong notes) and additions (extra notes) post-hoc. Wrong notes occur when a performer plays an incorrect note in place of a correct note, directly substituting the correct note for the incorrect note. Extra notes occur when a performer inserts an incorrect note between two correct notes.

To the extent that agency ambiguity is associated with a collective sense of error ownership and blurred self–other representations in internal models, behavioural and neural signatures of error processing may differ when playing in synchrony at the same pitch relative to different pitches. However, agency ambiguity was not expected to influence behavioural and neural indices prior to playing an incorrect note, consistent with the assumption that internal models allow the nervous system to detect self motor programming errors before movement execution is complete and sensory (i.e. auditory) feedback arrives. Pre-error slowing and the pre-ERN should therefore occur prior to self-produced errors (but not other-produced errors) as in previous studies of solo performance (Maidhof *et al.*, 2009, 2013; Ruiz *et al.*, 2009).

Agency ambiguity was expected to influence post-error responses to the extent that musical joint action involves control processes that utilize sensory feedback of self and other. Greater post-error slowing after self-produced errors and other-produced errors with same pitches than different pitches would indicate that the brain is not fully able to resolve the self–other distinction prior to movement execution. EEG modulations could pinpoint the neural origin of this effect in compromised use of error-related sensory feedback by a ‘self’ internal model manifest as the Pe (Nieuwenhuis *et al.*, 2001; Godefroid *et al.*, 2016) or an ‘other’ internal model manifest as the FRN (cf. Loehr *et al.*, 2013).

## Methods

### Participants

Participants were 48 skilled pianists (26 female) with an age range of 18–84 years (*M* = 31.6 years, s.d. = 16.5) and an average of 18.8 years of piano playing experience (s.d. = 15.75, range = 3–75, median = 13). Forty-one participants self-reported as right handed (five left handed and two ambidextrous). Pianists in 15 pairs were familiar with each other; the other nine pairs were unfamiliar. All participants reported normal hearing and gave informed consent to participate in the study. The experiment was approved by the local ethics committee and conducted according to the declaration of Helsinki.

### Materials

The musical materials consisted of six piano pieces based on technical exercises (see Supplementary Materials SM1B) with a range of 145–169 notes and an instructed tempo of 80 beats per minute. Participants each performed in separate booths on a separate Kurzweil SP2X keyboard set to the ‘Grand Piano’ setting and heard their own and their partner’s performances through EEG-compatible insert earphones (Etymotic Research, ER1). The audio was routed through a mixer (Behringer Xenyx 1002) to combine the keyboard outputs before being sent through the earphones to both participants simultaneously. There was no stereo separation of parts into left and right channels or panning across channels. Each ear received the same mix of sound output from both pianos. Custom-built devices converted the musical instrument digital interface (MIDI) signals from each keyboard into serial signals compatible with Presentation software (Neurobehavioural Systems, Inc.) for each player. A computer program written in Presentation software controlled the visual presentation of stimuli (the scores of the piano pieces) and metronome sounds, logged all MIDI values and onset timings of the keystrokes played by each participant and sent triggers to the two computers recording the EEG data. Stimuli (in music notation form) were visually presented on 24-inch BENQ monitors. A MOTU micro-lite MIDI device and a MacBook laptop computer were used to record both participants’ performances as separate tracks in a single file (using Reaper v5.04/x64 software). This was used only for measuring key-press velocity (not timing).

### Design and procedure

Each pair of participants played the piano pieces in synchrony, unimanually with the right hand, in two agency conditions in a within-subjects design. In the ambiguous agency condition, participants played the pieces together at the exact same pitch. In the unambiguous agency condition, participants played the pieces one octave apart (see Supplementary Materials Figure SM1 for pieces). As participants played the pieces, keystroke onset timing (MIDI note on messages), MIDI note numbers and EEG activity were recorded. Behavioural and neural responses to self-produced and other-produced errors were compared to correct keystrokes for each participant. IKIs and asynchronies were computed during data analysis.

Pairs of participants received the piano pieces (scores and recordings) 1 week before their scheduled experiment session to rehearse and memorise before the experiment. After arrival and EEG preparation, each participant was ushered into a separate EEG booth containing a piano keyboard. Thus, participants could not see or speak to each other throughout the experiment. Participants were instructed to focus on synchronicity and told to continue playing if either of them made an error. Participants were instructed to visually monitor their hands while playing (see Supplementary Materials SM1C). Each trial began with the visual presentation on the monitors of the score to be played. After 7 s, a metronome presented four ticks to set the target tempo—80 beats per minute (188 ms per inter-beat-interval)—after which participants were required to start playing. Trials lasted from 26 to 34 s, depending on piece length. Before starting the experiment, participants completed at least six practice trials to become familiar with the procedure and to ensure all pieces were memorised. One (randomly chosen) participant in each pair was responsible for starting each trial by pressing the lowest key on the keyboard for the duration of the experiment.

The combination of six pieces by two agency conditions resulted in 24 possible piece combinations (i.e. unambiguous upper octave, unambiguous lower octave, ambiguous upper/lower and ambiguous lower/upper for the six different pieces). There were 144 trials split into three blocks of 48 trials. The possible piece combinations were split into two groups of 12 and each group was randomised twice per block to reduce the chance of a participant consecutively playing the same exact piece. After each block, participants were given a break until both said they were ready to continue. The whole experiment, including set-up and questionnaires, took ∼2.5 h to complete.

### Behavioural data analyses

#### Interpersonal synchrony.

Given that the instructed goal of the task was to play duos in synchrony, interpersonal synchronization for each pair was assessed by analysing keystroke asynchronies between co-performers. These asynchronies were calculated for the error and correct sequences split by agency ambiguity. Three asynchrony measures were computed: median unsigned (i.e. absolute) asynchrony, the coefficient of variation of asynchronies (i.e. variability of signed asynchronies normalized by IKIs) and synchronization failures (i.e. percentage of asynchronies larger than 75 ms) (see Supplementary Materials SM1D). Keystroke velocity data were also analysed and are reported in the Supplementary Materials Section SM2C.

#### Inter-Keystroke Intervals (IKIs).

**Fig. 1.**
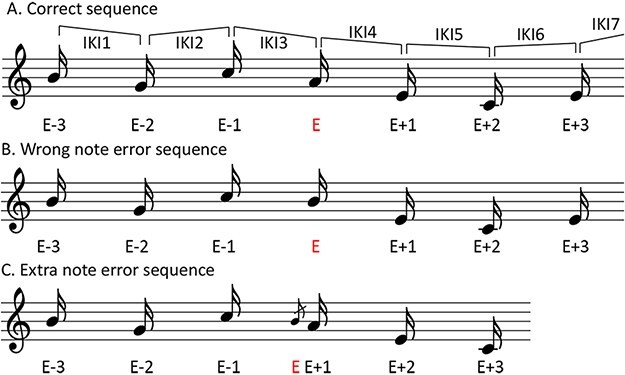
Examples of (A) correct note, (B) wrong note error and (C) extra note error sequences with labels. All sequences are based around the central note labelled E (in red), with three previous notes labelled E − 3, E − 2 and E − 1, and three following notes labelled E + 1, E + 2 and E + 3, respectively. IKI placements indicate the location of the IKI for each position.

Isolated pitch errors that were preceded and followed by three correct keystrokes (as in Ruiz *et al.*, 2009)—that is, response sequences of seven keystrokes with the error keystroke in the fourth position—were included in analyses. Keystrokes were labelled as follows: E−3, E−2, E−1, E, E + 1, E + 2, E + 3, with E being the error keystroke (see Figure 1 and Supplementary Materials SM1D for additional details). For each sequence, IKI was measured by subtracting the timing of the first keystroke from the second, the second from the third, and so on. An additional keystroke was included at the end of each sequence to provide seven IKI values. Response sequences were analysed separately for cases where the individual producing the IKIs made the error (self error) and for cases where the co-performer made the error (other errors). Only one participant from a pair had made an error in all cases. Each response sequence was thus assigned to two datasets, one containing self errors and one containing other errors, accounting for both individuals from each pair across data sets. These same procedures and classifications were used for the correct sequences. Partner sequences were identified by finding the keystroke corresponding to the self error keystroke (or correct keystroke, for self correct sequences) and ensuring that the three keystrokes before and after were correct in pitch and were under the timing criteria for inclusion in data analyses (see Supplementary Materials SM1D). All correct sequences during which the partner was also correct (but not when the partner made an error) were included in the analyses. Thus, there were more correct sequences than error sequences in the analyses.

One dataset (self error/correct) contained self-error and self-correct sequences, while the partner had performed correctly. Another dataset (other error/correct) contained self-correct sequences, while the partner had performed error sequences and correct sequences. In a first step, IKIs from both datasets were analysed using a 2×2 × 2 × 7 ANOVA (self/other × error/correct × agency [ambiguous/unambiguous] × interval position [IKI1-IKI7]). Results revealed that the differences between these conditions were driven by the self error condition (see Supplementary Materials SM2B for full analyses and figures). Thus, self and other data were analysed in separate 2 × 2 × 7 ANOVAs (error [error/correct] × agency [ambiguous/unambiguous] × interval position [IKI1-IKI7]) to focus on differences specifically for self and other. A Greenhouse–Geisser correction was applied when the degrees of freedom numerator exceeded 1. Because of the way IKI was calculated, the IKI terminated by the error keystroke is at interval position IKI3. In a second step, analyses were carried out on the self-produced and other-produced error data split by error type—extra note errors and wrong note errors. Extra note errors occur when a note not indicated in the score is inserted in the piece, usually with a very short IKI (Repp, 1996). Wrong note errors occur when an incorrect note is played instead of a correct note.

Data from four participants were excluded from the behavioural analyses because there were less than five errors in at least one condition for each participant. In addition, eight participants were excluded from analyses due to issues with the EEG data (see below). Thus, the IKI analyses for pooled error types were conducted on data from 36 participants. Additional participants were removed from the extra note versus wrong note split analysis because they made no wrong note errors, leaving 27 participants for those analyses.

### EEG data acquisition and analyses

Continuous EEG signals were recorded from 64 active electrodes placed over the scalp according to the extended 10–20 system. The data were re-referenced to the average of two electrodes placed over the left and right mastoids. A band-pass filter (0.5–30 Hz) was applied to the data to remove slow drifts and power line noise. The data were visually inspected, and trials containing technical and muscle artefacts (e.g. jaw movement) were removed. EEG data were epoched from 500 ms before error (or correct) onset to 1000 ms after error (or correct) onset and baseline corrected from 300 to 150 ms pre-error onset (as in Ruiz *et al.*, 2009) for self error/correct sequences and baseline corrected from 200 ms pre-error onset to 0 ms (i.e. at error onset) for other error/correct sequences. The epoched data were cleaned of eye blinks and saccade-related artefacts using independent component analysis (using the ‘runica’ algorithm, as implemented in Matlab toolboxes such as EEGLAB or Fieldtrip). For statistical analyses, electrodes were pooled (i.e. the average was computed) into nine regions of interest (ROIs) delineated by lateralisation (left, middle and right) and anterior/posterior (anterior, centre and posterior). Additional details of EEG data acquisition, ROIs and analyses are reported in the Supplementary Materials Section SM1E. The post-error time windows selected for analyses were 30–90 ms (ERN), 120–230 ms (Pe) and 215–300 ms (FRN). For analyses split by error type, there was an additional time window of 80–25 ms pre-error (pre-ERN) and the latency of the FRN shifted to 250–340 ms, specifically for extra note errors. The time windows for the ERN, FRN and pre-ERN were based on previous research on these components (Gehring *et al.*, 1993, 2012; Maidhof *et al.*, 2009, 2013; Ruiz *et al.*, 2009) with adjustments based on visual inspection of the current data. The time window for the Pe was selected because we observed an early, frontocentral Pe, but not a later, parietal Pe. Therefore, we used a latency that is consistent with research on the early Pe component (Van Veen and Carter, 2002; Ruchsow *et al.*, 2005; Ullsperger *et al.*, 2014b).

## Results

### Behavioural results

Descriptive statistics of errors by agency condition and error type are shown in Table 1. Isolated errors made up, on average, 0.29% of the total keystrokes played (s.d. = 0.35%). Of the isolated errors, an average of 23.94 (0.14%; s.d. = 0.16%) were committed in the ambiguous agency condition and 25.83 (0.15%; s.d. = 0.10%) in the unambiguous agency condition. The difference in amount of errors committed between conditions was not significant (*t*(35) = 1.538, *P* = 0.133). Extra note errors made up 0.16% (s.d. = 0.11%; unambiguous: 0.08%, s.d. = 0.06%; ambiguous: 0.08%, s.d. = 0.05%) and wrong note errors made up 0.18% (s.d. = 0.37%; unambiguous: 0.09%, s.d. = 0.2%; ambiguous: 0.08%; s.d. = 0.016%) of the isolated errors for the reduced number of participants in those analyses.

**Table 1.** Descriptive statistics for errors by agency conditions and error types

		Total	Percent	Mean	s.d.	Median	*n*
Agency conditions	Total keystrokes	722 195	–	20 060.97	1609.25	20 784	36
	Total errors	2077	0.29	57.69	70.07	34	36
	Ambiguous errors	995	0.14	27.64	32.67	16	36
	Unambiguous errors	1082	0.15	30.06	37.84	17	36
Error types	Total keystrokes	535 259	–	19 824.41	1695.71	20 220	27
	Extra note errors	902	0.17	33.41	22.43	25	27
	Wrong note errors	986	0.18	36.52	74.40	13	27

*Note*: Mean, median and s.d. are per participant (*n* = number of participants).

#### Interpersonal synchrony.

**Fig. 2.**
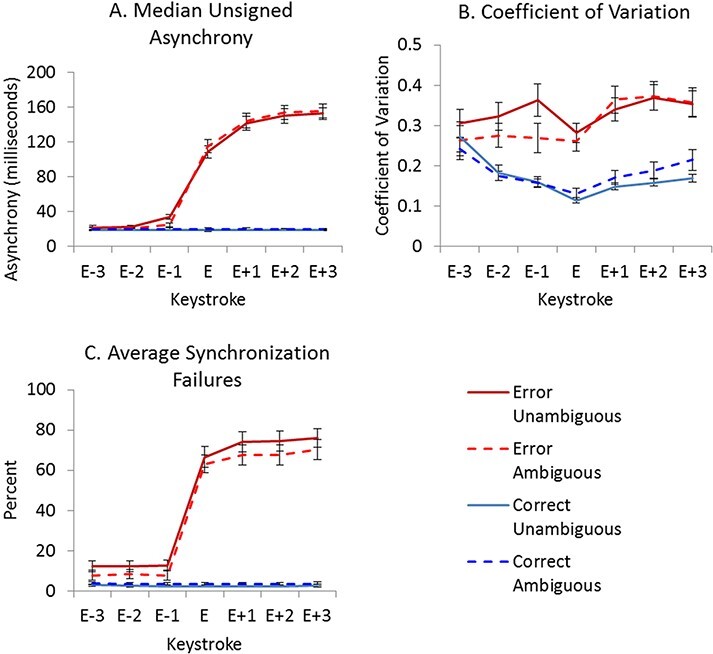
Asynchrony data associated with unambiguous and ambiguous agency conditions during error and correct sequences. (A) Median unsigned asynchrony averaged across participant pairs. (B) Coefficient of variation. (C) Mean synchronization failures. Error keystroke (and respective correct keystroke) is labelled E. Error bars show standard error.

To assess interpersonal synchrony between paired pianists, measures of keystroke asynchrony (i.e. median unsigned asynchrony, coefficient of variation and synchronization failures) were calculated for each pair for error and correct sequences in ambiguous and unambiguous agency conditions (see Figure 2). Full ANOVA results are included in the Supplementary Materials Section SM2A and only significant effects (*P* < 0.05) are reported below. Agency did not produce significant main effects or enter into significant interactions for unsigned asynchrony.

Figure 2A shows that, in sequences containing errors, unsigned asynchronies increased from the error keystroke onwards. A 2 × 2 × 7 ANOVA (error/correct × agency × keystroke) on the median unsigned asynchrony revealed a significant main effect of error/correct (*F*(1, 22) = 343.72, *P* < 0.001), a significant main effect of keystroke (*F*(1.48, 32.62) = 191.56, *P* < 0.001) and a significant interaction between error/correct and keystroke (*F*(1.51, 33.15) = 195.60, *P* < 0.001), but no significant main effect or interactions involving agency. Follow-up paired sample *t*-tests indicated that pianists were more asynchronous when playing error sequences than correct sequences at keystrokes E through E + 3 (all *P*-values < 0.001).

Coefficient of variation data (Figure 2B) show higher variability of asynchronies for error sequences than correct sequences already two keystrokes before the error position. The reliability of this result was confirmed in a 2 × 2 × 7 ANOVA (error/correct × agency × keystroke) showing significant main effects of error/correct (*F*(1, 22) = 33.08, *P* < 0.001) and keystroke (*F*(2.76, 60.71) = 11.67, *P* < 0.001) and significant interactions of error/correct × keystroke (*F*(2.20, 48.46) = 8.17, *P* = 0.001), as well as agency × keystroke (*F*(2.14, 47.07) = 3.95, *P* = 0.024). Follow-up *t*-tests for the error/correct × keystroke interaction indicated there was higher variability of asynchronies when playing error sequences than correct sequences on all except the first keystroke (all *P*-values < 0.001). Follow-up *t*-tests for the agency × keystroke interaction yielded no significant differences between ambiguous and unambiguous sequences at any keystroke.

#### Inter-Keystroke Intervals (IKIs).

Preliminary analyses of the IKI data for self and other combined datasets without splitting by error type are reported in the Supplementary Materials Section SM2B. Agency did not produce significant main effects or enter into significant interactions in these preliminary analyses. The IKI analyses reported here were conducted on data that were split by error type—extra note errors and wrong note errors. It should be noted that for extra note errors, the error and post-error keystrokes occurred within the time frame of a single correct keystroke. In other words, extra note errors most likely represent finger ‘slips’ that subdivide the intended IKI. Extra note errors were performed with an average IKI of 27.61 ms (s.d. = 45.43 ms) and post-error notes were performed with an average IKI of 123.05 ms (s.d. = 41.68 ms). The summed IKIs of E and E + 1 in the extra note sequences was, on average, 150.65 ms (s.d. = 33.48 ms), whereas the average IKI for correct keystrokes was 164.14 ms (s.d. = 22.41 ms). Ninety-nine percent of extra note errors were a neighbouring note (i.e. one keystroke up or down on the keyboard) from either the pre- or post-error keystroke.

**Fig. 3.**
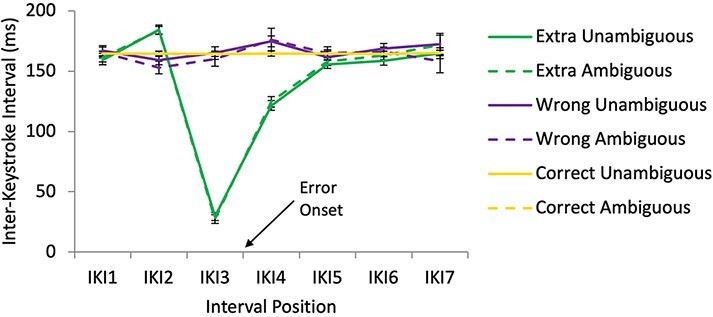
Self-produced IKI by error type and agency conditions—extra note errors and wrong note errors compared to correct note sequences. Onset of error keystroke is at the tick mark between IKI3 and IKI4. Errors bars show standard error.

Figure 3 shows the mean self-produced IKIs for individual notes during extra note errors, wrong note errors and correct sequences. It can be seen here that IKIs varied little across positions for all sequences apart from extra note error sequences. A 3×2 × 7 ANOVA (extra/wrong/correct × agency × interval position [IKI1–IKI7] revealed a significant main effect of extra/wrong/correct (*F*(1.41, 36.61) = 70.90, *P* < 0.001), a significant main effect of interval position (*F*(1.80, 46.68) = 56.09, *P* < 0.001) and a significant interaction between extra/wrong/correct and interval position (*F*(1.90, 49.27) = 62.03, *P* < 0.001), but no significant main effect or interactions involving agency (Supplementary Table SM28). Follow-up ANOVAs at the interval position level revealed a significant difference between extra, wrong and correct at IKI1, IKI2, IKI3 and IKI4 (Supplementary Table SM29).

Follow-up *t*-tests revealed that for self-produced extra note errors compared to wrong note errors, keystroke E-1 was late, as reflected by a larger value for IKI2, and keystrokes E and E + 1 were early, as reflected by smaller values for IKI3 and IKI4 (Table 2). Compared to correct notes, self-produced extra note errors were performed relatively late on keystroke E-1, as reflected by a larger value for IKI2, and early on keystrokes E and E + 1, as reflected by smaller values for IKI3 and IKI4. When playing wrong note errors compared to correct notes, participants performed keystroke E + 1 late, as reflected by a larger value for IKI4. With wrong note errors, post-error slowing was observed, but no pre-error speeding. Extra note errors showed results opposite of the expected pattern—pre-error slowing followed by error speeding and post-error speeding. With extra note errors, these effects occurred because the extra note subdivided an otherwise accurately timed interval between keystrokes E ‒ 1 and E + 1.

**Table 2.** *T*-test values for behavioural analysis of IKI between extra note error, wrong note error and correct sequences for positions where a significant main effect was found

	Extra vs. correct		Wrong vs. correct		Extra vs. wrong	
Position	*t*-value	*P*-value	*t*-value	*P*-value	*t*-value	*P*-value
IKI1	−1.24	0.225	1.73	0.096	−2.67	0.013
**IKI2**	**8.42**	**<0.001**	−2.62	0.014	**6.92**	**<0.001**
**IKI3**	**−35.00**	**<0.001**	0.01	0.995	**−24.32**	**<0.001**
**IKI4**	**−15.81**	**<0.001**	**2.73**	**0.011**	**−13.27**	**<0.001**

*Notes:* The error keystroke terminates IKI3 and initiates IKI4. Degrees of freedom = 26. Bold values indicate significant effects (Bonferroni corrected to *P* < 0.0125).

The 2 × 2 × 7 (error/correct × agency × interval position [IKI1—IKI7]) ANOVA on IKIs in the other-produced error dataset (i.e. IKIs of pianists in the context of error and correct sequences by their partners) yielded no significant main effects or interactions (Supplementary Table SM30). Thus, the performance timing of one pianist within a pair was not affected by errors produced by the other pianist. Analysis split by error type did not yield any significant main effects or interactions (all *P*-values > 0.1).

### EEG results

**Fig. 4.**
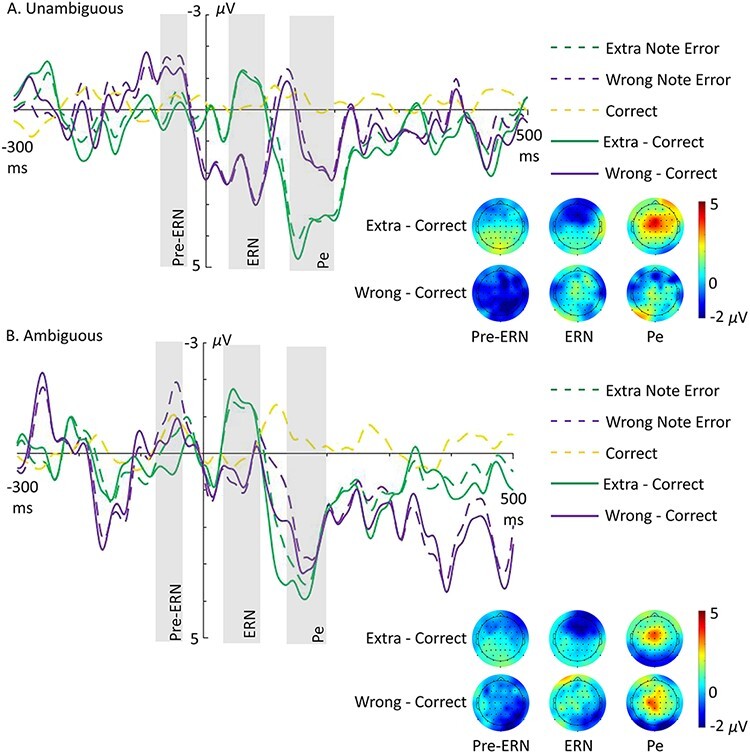
Grand-averaged waveforms showing pre-ERN (−80 to −25 ms), ERN (30–90 ms) and Pe (120–230 ms) time-locked to onset of self-produced extra note errors (green dashed), wrong note errors (purple dashed) and correct notes (yellow dashed) for unambiguous (A) and ambiguous (B) trials at electrode FCz. Solid lines show difference waves for extra note errors minus correct (green solid) and wrong note errors minus correct (purple solid). Shown below each waveform plot are the respective scalp voltage distributions for difference waves for pre-ERN (at −60 ms), ERN (at 60 ms) and Pe (at 150 ms) components. *NB*: Wrong note errors had a low number of trials.

**Fig. 5.**
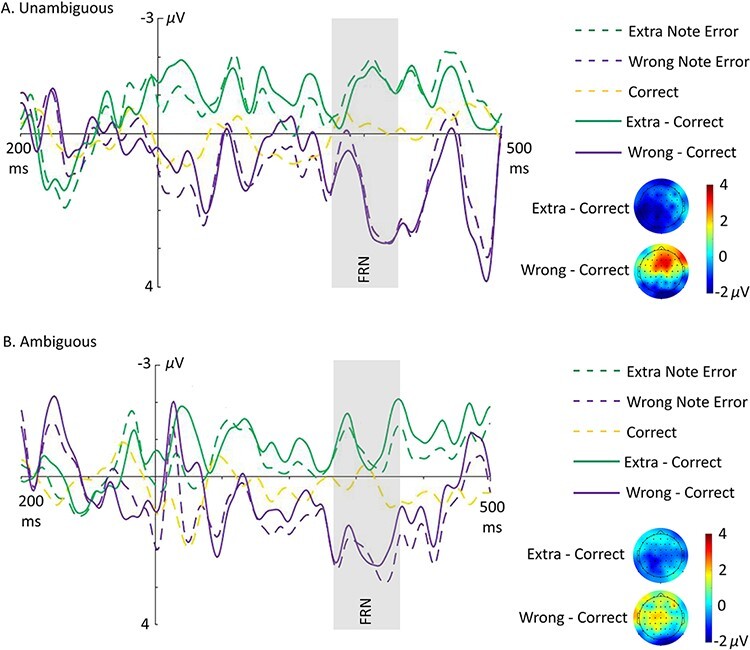
Grand-averaged waveforms showing FRN (250–340 ms) time-locked to onset of other-produced extra note errors (green dashed), wrong note errors (purple dashed) and correct notes (yellow dashed) for unambiguous (A) and ambiguous (B) trials at electrode Cz. Solid lines show difference waves for extra note errors minus correct (green solid) and wrong note errors minus correct (purple solid). Shown below each waveform plot are the respective scalp voltage distributions for difference waves for FRN (at 300 ms) components. *NB*: Wrong note errors had a low number of trials.

EEG waveforms and topographic maps are displayed for self error/correct data in Figure 4 and for other error/correct data in Figure 5. Preliminary analyses showed that self-produced wrong note errors were associated with a pre-ERN at a latency of −80 to −25 ms (i.e. before sound onset), while self-produced extra note errors elicited an ERN with a latency of 30–90 ms and a frontal distribution. Both of these components were followed by a fronto-central Pe with a latency of 120‒230 ms (see Supplementary Materials Section SM3A and SM3D). EEG data in these time windows were split by error type and analysed with 3 × 2 × 3 × 3 ANOVAs (extra/wrong/correct × agency × lateralisation [left/middle/right] × Anterior/Posterior [anterior/centre/posterior]). Other-produced errors elicited the FRN with a latency of 215–300 ms with a parietal distribution (see Supplementary Materials Section SM3F). When split by error type, the FRN was observed in the time window of 250–340 ms for extra note errors only. To anticipate the main outcome, no effects of agency were detected for any ERP, but novel and informative effects of error type were observed.

#### Error-Related Negativity (pre-ERN/ERN).

The extra/wrong/correct × agency × lateralisation × anterior/posterior ANOVA on the −80 to −25 ms pre-ERN time window for self error/correct data yielded a significant main effect of extra/wrong/correct (*F*(1.57, 40.87) = 4.48, *P* = 0.025), a significant interaction between extra/wrong/correct and lateralisation (*F*(2.87, 74.62) = 2.90, *P* = 0.043) and a significant interaction between extra/wrong/correct and anterior/posterior (*F*(2.17, 56.33) = 7.12, *P* = 0.001). There was no significant main effect of agency or interactions involving agency (Supplementary Table SM31). Breaking the ANOVA down by lateralisation revealed significant effects in all lateralised ROIs (see Supplementary Materials Section SM3B). Notably, ERP amplitude in the pre-ERN time window was significantly larger for wrong note errors and correct notes than extra note errors in the left and middle posterior ROIs and significantly larger for wrong note errors than extra note errors or correct notes in the right centre and posterior ROIs. In addition, we conducted ANOVAs to increase signal-to-noise ratio for detecting effects of agency: one ANOVA to identify ROIs in which EEG activity differed between error and correct responses regardless of agency and follow-up ANOVAs that included only self-produced error responses (i.e. without correct responses) separated between agency conditions in the relevant ROIs. These additional analyses revealed no such effects (Supplementary Tables SM32 and SM33).

The extra/wrong/correct × agency × lateralisation × anterior/posterior ANOVA on the 30–90 ms ERN time window for self error/correct data yielded a significant main effect of anterior/posterior (*F*(1.22, 31.63) = 4.22, *P* = 0.041), an interaction between extra/wrong/correct and anterior/posterior (*F*(1.70, 44.15) = 4.69, *P* = 0.019) and an interaction between extra/wrong/correct, lateralisation and anterior/posterior (*F*(3.67, 95.47) = 2.90, *P* = 0.03). There was no significant main effect of agency or interactions involving agency (Supplementary Table SM34). Follow-up tests revealed that ERP amplitude in the ERN time window was significantly larger for extra note errors than wrong note errors or correct notes in the middle anterior ROI (see Supplementary Materials Section SM3C). Supplementary ANOVAs to increase signal-to-noise ratio for detecting effects of agency did not reveal effects of agency (Supplementary Tables SM35 and SM36).

#### Error Positivity (Pe).

The extra/wrong/correct × agency × lateralisation × anterior/posterior ANOVA on the 120–230 ms Pe time window for self error/correct data revealed a significant main effect of lateralisation (*F*(1.93, 50.18) = 9.37, *P* < 0.001), a significant interaction between extra/wrong/correct and anterior/posterior (*F*(1.53, 39.73) = 5.40, *P* = 0.014) and a significant interaction between lateralisation and anterior/posterior (*F*(2.62, 68.19) = 6.14, *P* = 0.002). There was no significant main effect of agency or interactions involving agency (Supplementary Table SM37). Breaking the ANOVA down by anterior/posterior revealed significant effects in anterior, centre and posterior ROIs (see Supplementary Materials Section SM3E). The Pe was larger for extra note errors than correct in the middle anterior and centre ROIs and larger for wrong note errors than correct in the middle centre ROI. In posterior ROIs, amplitude was larger in the middle compared to the right and did not differ significantly between error types. Supplementary ANOVAs to increase signal-to-noise ratio for detecting effects of agency did not reveal effects of agency (Supplementary Tables SM38 and SM39).

#### Feedback-Related Negativity (FRN).

The extra/wrong/correct × agency × lateralisation × anterior/posterior ANOVA on the 250–340 ms FRN time window for other error/correct data yielded a significant main effect of extra/wrong/correct (*F*(1.8, 45.09) = 6.72, *P* = 0.004), a significant main effect of anterior/posterior (*F*(1.16, 28.91) = 6.53, *P* = 0.013) and an interaction between extra/wrong/correct and lateralisation (*F*(2.46, 61.51) = 4.47, *P* = 0.01). There was no significant main effect of agency or interactions involving agency (Supplementary Table SM40). Follow-up tests revealed that ERP amplitude in the FRN time window was significantly larger for extra note errors than wrong note errors in all ROIs except the right centre and right posterior. Amplitudes in this window were also larger for extra note errors than correct in the left and middle centre and posterior ROIs and for correct than for wrong note errors in the middle and right anterior and middle centre ROIs (see Supplementary Materials Section SM3G). Supplementary ANOVAs to increase signal-to-noise ratio for detecting effects of agency did not reveal effects of agency (Supplementary Tables SM41 and SM42).

## Discussion

The current experiment investigated behavioural and neural responses to naturally occurring errors in a musical joint action task that required precise real-time interpersonal coordination under conditions that varied in agency ambiguity. Accordingly, pairs of highly skilled pianists simultaneously played piano pieces in unison (ambiguous agency due to identical pitches) and octaves (unambiguous agency due to different pitches) as behavioural and neural measures were recorded. While the agency manipulation did not produce reliable effects on performance timing or ERPs, the results are informative about how the action system deals with different types of self- and other-produced errors.

### Overall effects of self and other errors

For self-produced errors, pianists showed a pattern of pre-error and error speeding. These behavioural responses were accompanied by an ERN peaking around 60 ms in the middle anterior ROI and a Pe peaking around 170 ms. Partner-produced errors did not affect self behaviour but did elicit an FRN peaking around 275 ms, consistent with previous research in joint action and error processing (Nieuwenhuis *et al.*, 2004; Loehr *et al.*, 2013; Huberth *et al.*, 2019).

Taken together, these results do not match predictions based on previous piano performance experiments with solo pianists (Maidhof *et al.*, 2009, 2013; Ruiz *et al.*, 2009) but are comparable to EEG results found in error processing for discrete tasks (Gehring *et al.*, 2012). Our results may differ from previous piano performance studies because solo playing is internally self-paced, allowing the performer greater freedom to adjust their timing to recover from an error. In contrast, timing in duo performance is constrained by the need for co-performers to maintain synchrony, which creates a situation where each individual is to some degree externally paced by the other, leaving little room for one individual to delay while the other forges ahead. Error management strategies, which are a vital component of skilled performance (Kruse-Weber and Parncutt, 2014), may thus differ for solo and ensemble performance. Further, in previous studies, an isolated error only needed to be preceded and followed by three correct keystrokes, whereas in the current study, there was the additional criterion that the partner’s concurrent performance needed to be error-free. Perhaps as a consequence of this, the current study had a slightly lower isolated error rate compared to previous studies (0.3% compared to 0.7% in Ruiz *et al.*, 2009).

Contrary to hypotheses, the agency manipulation did not reveal any significant results in the overall analyses for self-produced or other-produced errors. This might be in part due to greater focus of attention by co-performers on timing than pitch information, as sensorimotor synchronization primarily involves the alignment of sound onset timing (Snyder and Krumhansl, 2001; Prince and Pfordresher, 2012). Alternatively, lack of effects of the agency manipulation may indicate that expert pianists rely on proprioceptive and tactile feedback more than auditory feedback when monitoring their own performance (Finney, 1997; Repp, 1999; Maidhof *et al.*, 2009, 2013; Ruiz *et al.*, 2009; Van Der Steen *et al.*, 2014). If error processing is triggered by proprioceptive and tactile feedback, auditory feedback is redundant and the agency manipulation in pitch may not result in agency ambiguity.

Strong action–perception coupling and accurate yet flexible internal models may improve monitoring and prediction of a partner’s performance, increasing self–other distinction even under ambiguous agency conditions. Experts have increased ability to identify their own actions over the actions of others (Knoblich and Flach, 2003; Repp and Knoblich, 2004; Loula *et al.*, 2005) due to strong action-perception coupling (Zatorre *et al.*, 2007; Novembre and Keller, 2014) and accurate internal models (Wolpert *et al.*, 1995; Keller *et al.*, 2016) developed through extensive training. People are sensitive to subtle variations in timing of actions, allowing for identification of their own performances among those of others (Knoblich and Flach, 2001; Knoblich *et al.*, 2002; Flach, Knoblich, and Prinz, 2003; Flach *et al.*, 2004; Keller *et al.*, 2007). Further, previous experience at performing an action enables better simulation and prediction of those action outcomes (Knoblich *et al.*, 2002; Lahav *et al.*, 2007) and allows for increased prediction of those same actions being performed by another (Knoblich *et al.*, 2002). Thus, in highly skilled performers, error awareness remains consistent even when it is more difficult to distinguish between their own performance and a partner’s performance.

Still, there may be a distinction between objective and subjective aspects of the task. While objective behavioural and ERP measures indicated that co-performers were generally able to distinguish each other’s actions irrespective of agency ambiguity, subjective reports suggested that some individuals found it difficult. Specifically, when asked in the post-experiment questionnaire if they could hear and distinguish their own playing from their partner’s playing, 46% of participants responded ‘yes, all the time’, 15% responded ‘only when playing in octaves’ and the remaining 39% responded ‘no, not consistently’. These individual differences suggest that it might be useful in future work to investigate agency using differing degrees of pitch separation and/or differences in timbre.

### Effects of different error types

The current findings demonstrate that different types of self-produced errors are processed at different latencies in expert performers. Data split by error type—extra note errors and wrong note errors—revealed that the behavioural error speeding was driven by extra note errors and revealed differences in neural activity based on error type. Extra note errors were quickly corrected, displayed pre-error slowing, error and post-error speeding and were processed post-error. Wrong note errors were left uncorrected, demonstrated post-error slowing and displayed pre-error ERPs. Both error types show a different pattern than observed in previous piano performance experiments, which focussed exclusively on wrong note errors (Maidhof *et al.*, 2009; Ruiz *et al.*, 2009).

For sequences containing extra note errors, participants played the pre-error note slightly late and played the error note and post-error note early. By the post-error note, participants were back on pace with correct notes. These errors were almost always neighbouring notes on the keyboard, as is consistent with previous literature on errors in sequential performance (Palmer and Van De Sande, 1993; Palmer and Pfordresher, 2003; Pfordresher *et al.*, 2007). These errors were quickly corrected within the timeframe of a single keystroke at the target tempo, allowing the erring partner to maintain synchrony with their partner even while correcting the error. The context of a musical joint action task, where participants are required to stay in time with their partners, may have increased the likelihood of motor execution errors that minimally interfere with the timing of surrounding actions.

Extra note errors elicited the ERN peaking around 40 ms post-error. Amplitude of the ERN was larger when playing extra note errors than when playing wrong note errors or correct notes. As extra error notes were quickly followed by a correct note, the results are consistent with previous research showing that corrected errors elicit larger ERN amplitudes compared to uncorrected errors (Gehring *et al.*, 1993; Rodríguez-Fornells *et al.*, 2002; Fiehler *et al.*, 2004, 2005; Ullsperger *et al.*, 2014a; Kalfaoğlu *et al.*, 2018). Other-produced extra note errors elicited the FRN, whereas the wrong note errors did not.

These results partially support the conflict monitoring theory of error processing, suggesting that errors result from pre-response conflict (Carter *et al.*, 1998; Botvinick *et al.*, 2001, 2004). This theory posits that the ERN is elicited by conflict between multiple competing responses and that an error response reinforces the conflict, resulting in post-error slowing (Ullsperger *et al.*, 2014a). It is possible that, in the current experiment, the competing motor responses were developed in parallel internally, resulting in response conflict. Multiple internal models can be developed in parallel for highly trained actions (Wolpert and Kawato, 1998; Haruno *et al.*, 2001). These multiple models may result in response conflict if the performer is unsure of which response needs to be carried out next. Response conflict can lead to response inhibition, allowing for additional time to decide on the appropriate response (Frank, 2006; Brittain *et al.*, 2012). Thus, the pre-error slowing may be indicative of response conflict. Further, the automatic correction suggests that the correct motor response was available, just delayed. Although performing music that has been memorized is different than choosing an action depending on possible outcomes, hesitation due to a brief doubt or lapse in memory could be similar to hesitation related to conflict in choosing between possible outcomes. The conflict monitoring theory therefore provides a parsimonious account for both types of response uncertainty.

Alternatively, extra note errors could be action slips that are instances of biomechanical implementation failure (Heckhausen and Beckmann, 1990; Reason, 1990; Botvinick and Bylsma, 2005). This would suggest that the intention and planning leading up to the action were correct, but the action failed at implementation. As extra note errors were quickly corrected, it seems reasonable to assume that the correct intentions and motor plans were prepared prior to the execution of the actions, but an incorrect action was subsequently implemented. A faster tempo increases the incidence of errors in piano performance, that is, a speed-accuracy trade-off, lending weight to the biomechanical implementation failure explanation. A biomechanical implementation failure could occur within the framework of response conflict, so these explanations need not be mutually exclusive but may be interconnected. Future research may be able to disentangle errors involving response conflict and biomechanical implementation failures.

Wrong note errors, on the other hand, were associated with post-error slowing. Compared to correct and extra note error sequences, participants played the post-error note late in the wrong note error sequences. These wrong note errors are more similar to traditional errors showing post-error slowing (for a review, see Rabbitt, 1966; Debener *et al.*, 2005; Danielmeier and Ullsperger, 2011), in which an incorrect motor plan and command is carried out without any corrective responses to compensate for the error.

Wrong note errors elicited a pre-ERN peaking around 30 ms pre-error. The amplitude of the pre-ERN was the greatest in the posterior ROIs, especially on the right side. The latency of this component is comparable to that of the pre-ERN observed in previous piano studies on performance errors, which was interpreted as early error detection (Maidhof *et al.*, 2009, 2013; Ruiz *et al.*, 2009). In these previous studies, the pre-ERN was found in fronto-central regions. The main difference in our study which could explain the change in pre-ERN topography may be that performers were instructed to visually monitor their hands while playing, whereas in previous studies, performers were blocked from visual monitoring of their hands.

The comparison between wrong note errors and extra note errors in the current study provides additional insight into this interpretation. To the extent that a pre-ERN indicates early error detection, it should increase the chance for error correction. Instead, wrong note errors were left uncorrected and extra note errors were corrected. Error correction occurred within 30 ms of error commission. Given that the pre-ERN is normally observed ∼80 ms before the error is committed, this early error detection could allow for corrective action to override the error before it is committed. Because of this, it seems unlikely that the pre-ERN is related to error detection but may instead be an indicator of performance breakdown leading to an error.

Both the ERN and pre-ERN were followed by the early Pe peaking around 170 ms. These ERP patterns are consistent with errors committed during solo performance. Previous research has suggested that the Pe is related to error awareness (Nieuwenhuis *et al.*, 2001; Hewig *et al.*, 2011; Murphy *et al.*, 2012; Godefroid *et al.*, 2016). As extra note errors were quickly corrected, it is reasonable to assume that performers were aware of making these errors (based on proprioceptive and tactile feedback, in advance of auditory feedback), at least aware enough to correct the errors. Performers were also likely aware of the errors during wrong note sequences. Although we predicted that the effect of agency may be observed in differences in the Pe amplitude, no significant differences were found related to agency.

Responses to the error types were slightly different, but both can be accounted for by the adaptive orienting theory (Wessel and Aron, 2017; Wessel, 2018). The theory posits that unexpected events, such as errors, initiate a cascade of behavioural and neural responses, including global motor suppression, because the event results in outcomes different than predicted. Wrong note errors displayed post-error slowing and elicited the pre-ERN and Pe; thus, the error led to response suppression. Extra note errors demonstrated error and post-error speeding and elicited the ERN and Pe. The increased speed for extra note errors is related to the errors being corrected. Error-correcting responses are faster than equivalent correct responses (Rabbitt, 1966, 2002; Cooke and Diggles, 1984), and the first step of error correction is suppression (Cooke and Diggles, 1984). Further, it is likely that these corrections are developed internally in parallel with the errors (Rabbitt, 2002), allowing for the corrected response to be carried out immediately after an early error response (Wolpert and Kawato, 1998; Rodríguez-Fornells *et al.*, 2002; Ullsperger and Von Cramon, 2006; Yeung and Summerfield, 2012). These corrections can occur automatically and unreflectively (Rabbitt *et al.*, 1978; Yeung and Summerfield, 2012; Ullsperger *et al.*, 2014a). Thus, the adaptive orienting theory is supported by data related to errors that result from an incorrect motor command, such as pressing an incorrect key in the current study, and also by data from errors that result from response conflict and are followed by rapid correction.

## Conclusions

The current findings indicate that musicians can distinguish their own performance from a partner’s performance even when agency is rendered ambiguous based on pitch information. Skilled pianists spend years practising and monitoring their own playing. Thus, even when playing with a partner, performance monitoring levels are high and agency ambiguity does not necessarily affect their ability to monitor their own or their partner’s performance. Additional research is needed to determine how agency ambiguity affects performance in joint action more generally, but the ambiguity may need to be in a dimension that is prioritized within the task (e.g. a rhythmic tapping task that prioritizes synchronicity). Different types of self-produced errors were processed in different ways. Through extensive training, skilled individuals develop an action control system supporting fluent interpersonal coordination by invoking distinct neural mechanisms to manage different types of errors.

## Supplementary Material

nsab019_Supp
