# Supplementary material for: Not all errors are alike: modulation of error-related neural responses in musical joint action
Source: Soc Cogn Affect Neurosci. 2021 Feb 10;16(5):512–24. doi: 10.1093/scan/nsab019 (PMC8094995; doi:10.1093/scan/nsab019)
Supplement: nsab019_Supp [file nsab019_supp.zip › scan-20-114-File008.docx]

**Supplementary Materials**

**SM1: Methods**

**SM1A: *Participants***

Participants had a minimum of 5^th^ Grade in the Australian Music Education Board qualification system or equivalent.

**SM1B: *Materials***

Piano pieces were modified versions of piano exercises by Charles-Louis Hanon (Hanon, 1923; see Figure SM1). Each participant received two sets of piano pieces with the second set containing the same six pieces one octave lower than the first set.

The following self-report questionnaires were employed to measure personality and social factors that have been shown to be related to music performance, inter-personal coordination, or joint action: the Liebowitz Social Anxiety Scale (Baker, Heinrichs, Kim, & Hofmann, 2002; Varlet et al., 2014), the Interpersonal Reactivity Index (Davis, 1980; Novembre, Ticini, Schütz-Bosbach, & Keller, 2012), the Big Five Inventory (10-item version; Luck, Saarikallio, Burger, Thompson, & Toiviainen, 2010; Rammstedt & John, 2007), the Core Self-Evaluations Scale (Judge, Erez, Bono, & Thoresen, 2003), and the IPC Locus of Control questionnaire (Fairhurst, Janata, & Keller, 2014; Levenson, 1973). These questionnaires made up the first part of the questionnaire for this experiment. The second part of the questionnaire consisted of questions related to participants’ performance in the experiment, their partner’s performance, their music practice habits, how much they prepared for the experiment, and if they were familiar with their partner. As they were being prepared with an EEG cap, participants completed the first part of the questionnaire. Participants completed the second part of the questionnaire after completing the experimental trials.

**SM1C: *Design and Procedure***

Participants were instructed to visually monitor their hands throughout the experiment. This was to prevent excessive eye movement and head movement which would produce artefacts in the EEG data. Additionally, if participants made an error and had to stop playing for a few keystrokes, visually monitoring their hands facilitated joining in again with their partner.

**SM1D: *Behavioural Data Analyses***

An algorithm was developed in MatLab comparing the MIDI performances with the score templates to locate errors in the performance. To maintain as little variation in timing as possible, only isolated pitch errors were included in the analysis.

For each sequence, IKI was measured by subtracting the timing of the first keystroke from the second, the second from the third, and so on. Thus, the error keystroke terminates IKI3 and initiates IKI4.

Interpersonal asynchronies were also analysed to assess overall synchronization performance. If keystrokes were more than 75 ms apart from the partner’s keystrokes, these were removed from the analysis (and counted as synchronization failures), as they would no longer be sufficiently synchronous with their partner. The rationale for this criterion was as follows. Preliminary analyses revealed that correct keystrokes were played with an average IKI of 164.14 ms (SD = 22.41 ms). One standard deviation less is 141.72 ms and half of that is 70.86 ms. Thus, keystrokes played more than 75 ms apart from a partner’s keystroke could be landing between the keystrokes of the partner instead of in synchrony with them. With these restrictions, there was an average of 49.77 errors per participant (SD = 64.15) out of an average of 20096.19 keystrokes played (SD = 1619.27 keystrokes). The same restrictions and procedure was done for correct keystroke sequences.

For the remaining asynchronies, measures of synchronization accuracy and variability were computed for each sequence. For synchronization accuracy, median unsigned asynchrony was calculated by taking the absolute value of the difference in keystroke onset times for each pair of corresponding keystrokes within a pair. Synchronization variability was assessed by computing the coefficient of variation of asynchronies, that is, the standard deviation of signed asynchronies (pianist 1 keystroke onset times minus pianist 2 keystroke onset times) for each sequence, divided by the mean IKI (averaged across pianists) for the sequence.

For keystroke velocity, data were not recorded properly for ten participants due to a technical problem. Thus, the velocity analyses were conducted on data from 27 participants.

**SM1E: *EEG data acquisition and analyses***

Continuous EEG signals were recorded from 64 Ag/AgCl electrodes placed over the scalp according to the extended 10-20 system (FPZ, FP1, FP2, AFZ, AF3, AF4, AF7, AF8, FZ, F1, F2, F3, F4, F5, F6, F7, F8, FCZ, FC1, FC2, FC3, FC4, FC5, FC6, FT7, FT8, CZ, C1, C2, C3, C4, C5, C6, T7, T8, CPZ, CP1, CP2, CP3, CP4, CP5, CP6, TP7, TP8, PZ, P1, P2, P3, P4, P5, P6, P7, P8, P9, P10, POZ, PO3, PO4, PO7, PO8, OZ, O1, O2, IZ), referenced to linked mastoids (M1, M2). The signals were acquired using a 24-bit BioSemi Active Two system (BioSemi B. V., Amsterdam, Netherlands). Vertical electrooculograms were recorded from the outer canthus of each eye and horizontal electrooculograms were recorded from above and below the right eye for each participant. Impedances were kept below 5 kΩ. Data were sampled at 512 Hz. We used FieldTrip MatLab Toolbox (Oostenveld, Fries, Maris, & Schoffelen, 2011) for data processing and visualisation. A band-pass filter (0.5 Hz – 30 Hz) was applied to the data to remove slow drifts and power line noise. The data were visually inspected and trials containing technical and muscle artefacts (e.g., jaw movement) were removed. The data were cleaned of eye blinks and horizontal eye movements using independent component analysis (ICA). EEG data were epoched from 500 ms before error (or correct) onset to 1 s after error (or correct) onset and baseline corrected from 300 ms to 150 ms pre-error onset (as done in Ruiz, Jabusch, & Altenmüller, 2009) for self error/correct sequences and baseline corrected from 200 ms pre-error onset to 0 ms (i.e., at error onset) for other error/correct sequences. For two participants, the EEG activity was only recorded for part of the experiment and thus was not included in the analysis. Data from another two participants were removed due to overly noisy EEG signal and a high level of artefacts throughout the experiment. Data from an additional eight participants were removed due to having less than 15 isolated errors to analyse. With these exclusions, data from a total of 36 participants were included in the overall EEG analysis, 27 participants in the extra/wrong note errors analysis, and 22 participants for the velocity extra/wrong note errors analysis.

Analyses of variance (ANOVAs) were conducted on the mean amplitudes in each condition in specific time windows for self errors and other errors with electrodes pooled into nine regions of interest (ROI), split by laterality and anterior/posterior location. Greenhouse-Geisser corrections were applied when the degrees of freedom numerator exceeded one in all analyses. The ROIs were delineated as follows: left anterior – F3, F5, F7, FC3, FC5, FT7; left centre – C3, C5, T7, CP3, CP5, TP7; left posterior – P3, P5, P7, PO3, PO7; middle anterior – F1, Fz, F2, FC1, FCz, FC2; middle centre – C1, Cz, C2, CP1, CPz, CP2; middle posterior – P1, Pz, P2, POz; right anterior – F4, F6, F8, FC4, FC6, FT8; right centre – C4, C6, T8, CP4, CP6, TP8; and right posterior – P4, P6, P8, PO4, PO8 (Sammler, Novembre, Koelsch, & Keller, 2013).

**SM2: Behavioural Results**

The expected number of keystrokes per participant, if all trials were played perfectly, was 21264. On average, participants played 20096.19 keystrokes (SD = 1619.27) and committed 49.77 isolated errors (SD = 64.15). The reduced number of keystrokes was observed because participants often missed keystrokes after an error. During some trials, when a mistake was made, participants could not start playing again for a few seconds, or sometimes not until the next trial. These errors did not occur in any consistent manner (i.e., not for one specific piano piece over or more than any others).

**SM2A: *Interpersonal synchrony***

A 2 x 2 x 7 ANOVA (Error/Correct x Agency x Keystroke) on the average synchronization failures revealed a significant main effect of Error/Correct (*F*(1, 22) = 309.17, *p* < 0.001), a significant main effect of Keystroke (*F*(1.15, 25.28) = 127.96, *p* < 0.001), and a significant interaction between Error/Correct and Keystroke (*F*(1.15, 25.24) = 126.11, *p* < 0.001). Full ANOVA results are shown in Table SM1. Follow-up *t*-tests showed that pairs had greater synchronization failures when playing error sequences than when playing correct sequences at keystrokes E through E+3 (all *p*-values < 0.001). There were no main effects or interactions involving Agency.

Table SM1
*ANOVA values for behavioural analysis of synchronization failures at each keystroke with factors of Error/Correct x Agency x Keystroke*

| Effect | *df* | *F* | *p*-value | *η_p_^2^* |
| --- | --- | --- | --- | --- |
| **E/C** | **(1, 22)** | **309.17** | **< 0.001** | **0.934** |
| Agency | (1, 22) | 1.60 | 0.219 | 0.068 |
| **Keystroke** | **(1.15, 25.28)** | **127.96** | **< 0.001** | **0.853** |
| E/C x Agency | (1, 22) | 3.02 | 0.096 | 0.121 |
| **E/C x Keystroke** | **(1.15, 25.24)** | **126.11** | **< 0.001** | **0.851** |
| Agency x Keystroke | (1.38, 30.43) | 0.26 | 0.689 | 0.012 |
| E/C x Agency x Keystroke | (1.39, 30.55) | 0.30 | 0.66 | 0.014 |

*Note:* Bold values indicate significant effects (*p* < 0.05). E/C = Error/Correct.

When split by error type, the asynchrony data show a distinct difference between the error types (see Figure SM2). Analyses of this data did not include the Agency factor because there were too few wrong note errors to split into agency conditions when looking at the data by pairs. It should be noted that the wrong note data were mostly supplied by two pairs, as that data accounted for 68 percent of the observations of wrong note errors for the asynchrony analyses. Additionally, the timing for extra note errors was adjusted to reflect the pitch-matched keystrokes. That is, since the extra note errors were very quick notes inserted into an otherwise correct sequence, the asynchrony calculations were adjusted so that the extra note was treated as an extra note. The differences in timing between partners was calculated as usual for the first three keystrokes, but skipped the extra note error to calculate asynchrony with the three post-error keystrokes, as those were more closely matched in time and matched in pitch between the partners. Thus, these sequences show six keystrokes instead of the previous sequences that showed seven keystrokes.


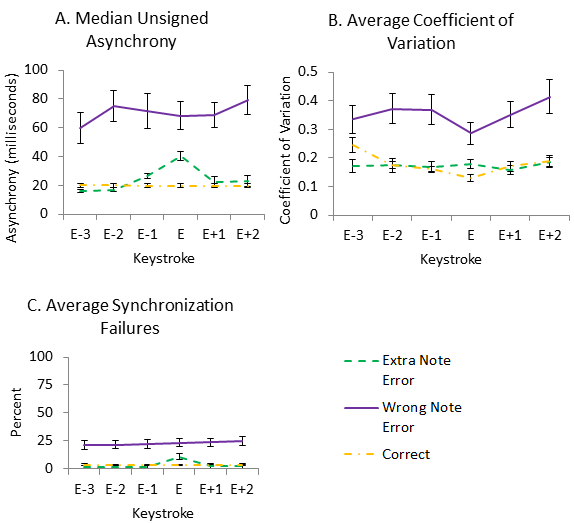


Figure SM2. Asynchrony data associated with extra note errors and wrong note errors during error and correct sequences. A. Median unsigned asynchrony averaged across pairs. B. Coefficient of variance. C. Mean synchronization failures. For wrong note and correct sequences, the error keystroke (and respective correct keystroke) is labelled E. For extra note error sequences, E represents the post-error note. Error bars show standard error.

A 3 x 6 ANOVA (Extra/Wrong/Correct x Keystroke) of the median unsigned asynchrony values revealed a significant main effect of Extra/Wrong/Correct (*F*(1.08, 21.30) = 25.69, *p* < 0.001), a significant main effect of Keystroke (*F*(3.53, 74.13) = 3.74, *p* = 0.011), and a significant interaction between Extra/Wrong/Correct and Keystroke (*F*(4.40, 92.30) = 3.95, *p* = 0.004). Follow-up ANOVAs at the Keystroke level revealed a significant difference between extra note errors, wrong note errors, and correct keystrokes at every keystroke location (see Table SM2 for full results).

Table SM2
*ANOVA values for behavioural analysis of median unsigned asynchrony at each keystroke with factors of Extra Note Error/Wrong Note Error/Correct Note.*

| Effect at each keystroke | *df* | *F* | *p*-value | *η_p_^2^* |
| --- | --- | --- | --- | --- |
| **E-3** | **(1.01, 21.30)** | **15.75** | **0.001** | **0.429** |
| **E-2** | **(1.02, 21.48)** | **29.85** | **< 0.001** | **0.587** |
| **E-1** | **(1.08, 22.69)** | **15.47** | **0.001** | **0.424** |
| **E** | **(1.17, 24.66)** | **15.22** | **< 0.001** | **0.420** |
| **E+1** | **(1.19, 24.95)** | **24.02** | **< 0.001** | **0.534** |
| **E+2** | **(1.19, 25.00)** | **27.44** | **< 0.001** | **0.566** |

*Note:* Bold values indicate significant effects (*p* < 0.05).

Finally, follow-up *t­*-tests (see Table SM3) showed that the median unsigned asynchrony was significantly lower at keystroke E-3 during extra note error sequences than during correct sequences and significantly higher at keystroke E (the post-error keystroke in the extra note sequences) in the extra note error sequences than the correct sequences. Pairs were more synchronous at the beginning of extra note error sequences than correct sequences, but less synchronous on the keystroke following an extra note error compared to a correct keystroke. When comparing wrong note error sequences to correct sequences, median unsigned asynchrony was significantly higher for all keystrokes during wrong note errors sequences. Pairs played wrong note error sequences with consistently less synchrony than correct sequences. When comparing extra note error sequences to wrong note error sequences, median unsigned asynchrony was significantly higher during wrong note error sequences for all keystrokes except the error keystroke in the wrong note error sequences and the post-error keystroke in extra note error sequences. Pairs played wrong note error sequences with less synchrony than extra note error sequences except when playing the error itself.

Table SM3
*T-test values for behavioural analysis of median unsigned asynchrony between extra note errors, wrong note errors, and correct notes at keystrokes. Degrees of freedom are (1, 21).*

|  | Extra vs. Correct | | Wrong vs. Correct | | Extra vs. Wrong | |
| --- | --- | --- | --- | --- | --- | --- |
| Keystroke | *t*-value | *p*-value | *t*-value | *p*-value | *t*-value | *p*-value |
| E-3 | **-4.17** | **< 0.001** | **3.80** | **0.001** | **-4.12** | **< 0.001** |
| E-2 | -2.88 | 0.009 | **5.33** | **< 0.001** | **-5.62** | **< 0.001** |
| E-1 | 2.36 | 0.028 | **4.27** | **< 0.001** | **-3.65** | **0.001** |
| E | **5.13** | **< 0.001** | **5.04** | **< 0.001** | -2.52 | 0.02 |
| E+1 | 0.66 | 0.518 | **5.82** | **< 0.001** | **-4.55** | **< 0.001** |
| E+2 | 0.58 | 0.566 | **6.24** | **< 0.001** | **-4.87** | **< 0.001** |

*Note:* Bold values indicate significant effects (Bonferroni corrected to *p* < 0.00278).

**SM2B: *Inter-Keystroke Intervals (IKIs)***

Figure SM3 shows the mean IKI for individual notes during sequences produced by participants (A) or by their partner (B) for both error sequences and correct sequences. A preliminary 2 x 2 x 2 x 7 (Self/Other x Error/Correct x Agency x Interval Position [IKI1-IKI7]) ANOVA showed significant main effects of Self/Other, Error/Correct, and Interval Position, and significant interactions for every combination of factors (*p* < 0.001 in all cases), with the exception of Agency and any interactions including Agency (*p* > 0.15 in all cases). Follow-up t-tests showed a significant difference between self error and self correct (*t*(1, 35) = 11.348, *p* < 0.001), and a significant difference between self error and other error (*t*(1, 35) = 12.55, *p* < 0.001), but no significant difference between self correct and other correct or other error and other correct (*p* > 0.2 in both cases). The similarity between the other error, other correct, and self correct data can be seen in Figure SM3a and b. Self-error IKI data were analysed with a 2 x 2 x 7 (Error/Correct x Agency x Interval Position [IKI1 – IKI7]) ANOVA. This analysis yielded a significant main effect of Error/Correct (*F*(1, 35) = 119.5, *p* < 0.001), a significant main effect of Interval Position (*F*(1, 35) = 152.74, *p* < 0.001), and a significant interaction of Error/Correct and Interval Position (*F*(1.89, 66.21) = 151.49, *p* < 0.001). Full ANOVA results are shown in Table SM4.


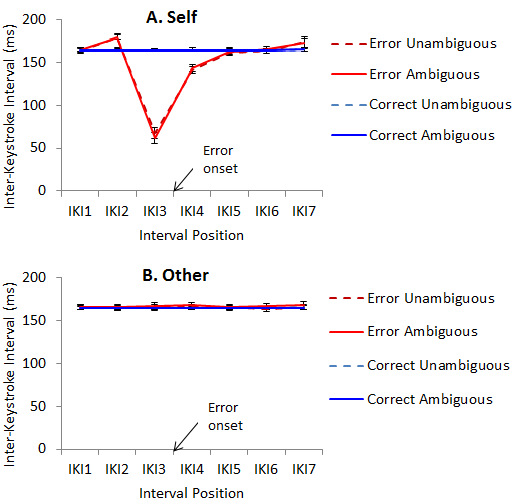


Figure SM3. Inter-keystroke intervals for error and correct sequences for: A. keystrokes produced by self, and B. keystrokes produced by self when other played error and correct sequences. Onset of the error keystroke is at the tick mark between IKI3 and IKI4. Error bars show standard error.

Table SM4
*ANOVA values for behavioural analysis of self IKI with factors of Error/Correct x Agency x Interval Position.*

| Effect | *df* | *F* | *p*-value | *η_p_^2^* |
| --- | --- | --- | --- | --- |
| **E/C** | **(1, 35)** | **119.50** | **< 0.001** | **0.773** |
| Agency | (1, 35) | 0.001 | 0.973 | 0.000 |
| **Position** | **(1.91, 66.92)** | **152.74** | **< 0.001** | **0.814** |
| E/C x Agency | (1, 35) | 0.01 | 0.938 | 0.000 |
| **E/C x Position** | **(1.89, 66.21)** | **151.49** | **< 0.001** | **0.812** |
| Agency x Position | (2.24, 78.39) | 0.89 | 0.426 | 0.025 |
| E/C x Agency x Position | (2.25, 78.82) | 0.84 | 0.446 | 0.024 |

*Note:* Bold values indicate significant effects (*p* < 0.05). E/C = Error/Correct.

Follow-up *t*-tests showed that IKI2 was significantly longer during error sequences than during correct sequences and that IKI3 and IKI4 were significantly shorter during error sequences than during correct sequences (see Table SM5 for statistical values and Figure SM3a). Although post-error slowing was predicted based on previous studies (for a review, see Danielmeier & Ullsperger, 2011), we observed pre-error slowing, error speeding, and post-error speeding. Participants played keystroke E-1 late, as reflected by the larger IKI2, in the error sequences than the corresponding keystroke in the correct sequences, and keystrokes E and E+1 early in the error sequences than in the corresponding correct keystrokes, as reflected by smaller IKI values at IKI3 and IKI4 (see Table SM5 and Figure SM3a). There were no significant effects or interactions involving agency.

Table SM5
*T-test values for behavioural analysis of IKI between error and correct sequences. Onset of error keystroke is between IKI3 and IKI4.*

| Interval Position | *df* | *t*-value | *p*-value |
| --- | --- | --- | --- |
| IKI1 | (1, 35) | -0.46 | 0.652 |
| **IKI2** | **(1, 35)** | **7.66** | **< 0.001** |
| **IKI3** | **(1, 35)** | **-15.66** | **< 0.001** |
| **IKI4** | **(1, 35)** | **-8.59** | **< 0.001** |
| IKI5 | (1, 35) | -2.11 | 0.042 |
| IKI6 | (1, 35) | -0.07 | 0.946 |
| IKI7 | (1, 35) | 1.84 | 0.075 |

*Note:* Bold values indicate significant effects (Bonferroni corrected to *p* < 0.007).

**SM2C: *Velocity***

Figure SM4 shows the mean keystroke velocity for individual notes during sequences produced by participants (A) or by their partner (B) for both error sequences and correct sequences. For each sequence, keystroke velocity was analysed by looking at MIDI velocity. Self keystroke velocity (i.e., keystroke velocity produced by the self during own performance) was analysed with a 2 x 2 x 7 (Error/Correct x Agency x Keystroke) ANOVA on self velocity showed a main effect of Error/Correct (*F*(1, 26) = 98.13, *p* < 0.001), a significant main effect of Keystroke (*F*(2.79, 72.59) = 82.28, *p* < 0.001), and a significant interaction between Error/Correct and Keystroke (*F*(2.81, 72.94) = 79.13, *p* < 0.001). Full ANOVA results are shown in Table SM6.


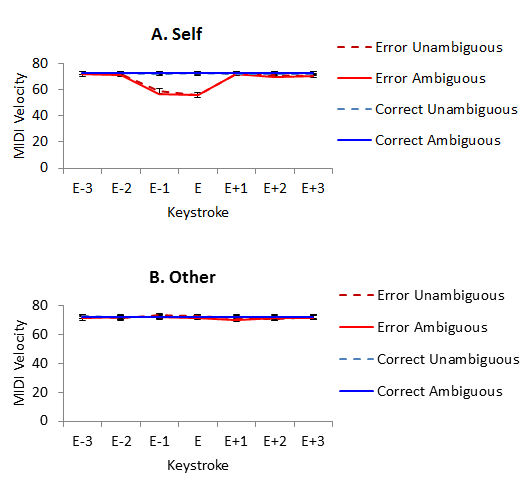


Figure SM4. Keystroke velocity for error and correct sequences for: A. keystrokes produced by self, and B. keystrokes produced by self when other played error and correct sequences. Error keystroke is labelled E. Error bars show standard error.

Table SM6
*ANOVA values for behavioural analysis of self velocity with factors of Error/Correct x Agency x Keystroke.*

| Effect | *df* | *F* | *p*-value | *η_p_^2^* |
| --- | --- | --- | --- | --- |
| **E/C** | **(1, 26)** | **98.13** | **< 0.001** | **0.791** |
| Agency | (1, 26) | 0.52 | 0.479 | 0.019 |
| **Keystroke** | **(2.79, 72.59)** | **82.28** | **< 0.001** | **0.760** |
| E/C x Agency | (1, 26) | 2.42 | 0.132 | 0.085 |
| **E/C x Keystroke** | **(2.81, 72.94)** | **79.13** | **< 0.001** | **0.753** |
| Agency x Keystroke | (3.81, 99.11) | 1.05 | 0.384 | 0.039 |
| E/C x Agency x Keystroke | (3.89, 101.16) | 1.07 | 0.375 | 0.039 |

*Note:* Bold values indicate significant effects (*p* < 0.05). E/C = Error/Correct.

Follow-up *t*-tests showed a significant difference in velocity between error and correct keystrokes on keystrokes E-1, E, and E+3 (see Table SM7 for statistical values and Figure SM4a) As predicted, participants played pre-error keystrokes and error keystrokes with less velocity than correct keystrokes, as shown by smaller velocity values on keystrokes E-1 and E. There were no significant effects or interactions involving agency.

Table SM7
*T-test values for behavioural analysis of self velocity between error and correct keystrokes.*

| Keystroke | *df* | *t*-value | *p*-value |
| --- | --- | --- | --- |
| E-3 | (1, 26) | -1.89 | 0.07 |
| E-2 | (1, 26) | -1.54 | 0.135 |
| **E-1** | **(1, 26)** | **-10.01** | **< 0.001** |
| **E** | **(1, 26)** | **-12.13** | **< 0.001** |
| E+1 | (1, 26) | -0.43 | 0.669 |
| E+2 | (1, 26) | -2.38 | 0.025 |
| **E+3** | **(1, 26)** | **-3.71** | **0.001** |

*Note:* Bold values indicate significant effects (Bonferroni corrected to *p* < 0.007).

To further investigate the pre- and post-error responses, analyses were conducted splitting the errors by type – extra note errors and wrong note errors. Figure SM5 shows the mean keystroke velocity for individual notes during sequences produced by participants for extra note error sequences, wrong note error sequences, and correct sequences. A 3 x 2 x 7 ANOVA (Extra/Wrong/Correct x Agency x Keystroke) found a main effect of Extra/Wrong/Correct (*F*(1.75, 36.72) = 31.65, *p* < 0.001), a main effect of Keystroke (*F*(3.07, 64.46) = 42.08, *p* < 0.001), and an interaction between Extra/Wrong/Correct and Keystroke (*F*(5.17, 108.62) = 28.97, *p* < 0.001). Full ANOVA results are shown in Table SM8.


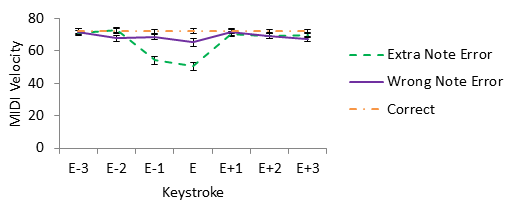


Figure SM5. Keystroke velocity by error type – extra note error sequences and wrong note error sequences compared to correct sequences. Error keystroke is labelled E. Errors bars show standard error.

Table SM8
*ANOVA values for behavioural analysis of self velocity with factors of Extra Note Error/Wrong Note Error/Correct Note x Agency x Keystroke.*

| Effect | *df* | *F* | *p*-value | *η_p_^2^* |
| --- | --- | --- | --- | --- |
| **E/W/C** | **(1.75, 36.72)** | **31.65** | **< 0.001** | **0.601** |
| Agency | (1, 21) | 0.05 | 0.821 | 0.002 |
| **Keystroke** | **(3.07, 64.46)** | **42.08** | **< 0.001** | **0.667** |
| E/W/C x Agency | (1.74, 36.59) | 0.45 | 0.615 | 0.021 |
| **E/W/C x Keystroke** | **(5.17, 108.62)** | **28.97** | **< 0.001** | **0.580** |
| Agency x Keystroke | (3.70, 77.70) | 0.96 | 0.428 | 0.044 |
| E/W/C x Agency x Keystroke | (4.40, 92.41) | 0.81 | 0.532 | 0.037 |

*Note:* Bold values indicate significant effects (*p* < 0.05). E/W/C = Extra Error/Wrong Error/Correct.

Follow-up ANOVAs at the Keystroke level revealed a significant difference between Extra, Wrong, and Correct on keystrokes E-2 (*F*(1.24, 26.07) = 10.99, *p* = 0.002), E-1 (*F*(1.72, 36.05) = 64.98, *p* < 0.001), E (*F*(1.63, 34.27) = 54.63, *p* < 0.001), and E+3 (*F*(1.27, 26.74) = 12.97, *p* = 0.001). Full ANOVA results are shown in Table SM9. When playing extra note errors compared to playing wrong note errors, participants played keystroke E-2 with more velocity and keystrokes E-1 and E with less velocity (see Table SM10 for statistical values and Figure SM5). When compared to correct notes, extra note errors were performed with less velocity on keystrokes E-1, E, and E+3. When playing wrong note errors compared to correct notes, participants performed keystrokes E-2, E, and E+3 with less velocity (see Table SM10 and Figure SM5).

Table SM9
*ANOVA values for behavioural analysis of self velocity at each keystroke with factors of Extra Note Error/Wrong Note Error/Correct Note.*

| Effect at each keystroke | *df* | *F* | *p*-value | *η_p_^2^* |
| --- | --- | --- | --- | --- |
| E-3 | (1.61, 33.90) | 1.10 | 0.343 | 0.05 |
| **E-2** | **(1.24, 26.07)** | **10.99** | **0.002** | **0.344** |
| **E-1** | **(1.72, 36.05)** | **64.98** | **< 0.001** | **0.756** |
| **E** | **(1.63, 34.27)** | **54.63** | **< 0.001** | **0.722** |
| E+1 | (1.28, 26.77) | 0.72 | 0.438 | 0.033 |
| E+2 | (1.33, 27.87) | 2.38 | 0.127 | 0.102 |
| **E+3** | **(1.27, 26.74)** | **12.97** | **0.001** | **0.382** |

*Note:* Bold values indicate significant effects (*p* < 0.05).

Table SM10
*T-test values for behavioural analysis of keystroke velocity between extra note errors, wrong note errors, and correct notes at keystrokes where a significant main effect was found. Degrees of freedom are (1, 21).*

|  | Extra vs. Correct | | Wrong vs. Correct | | Extra vs. Wrong | |
| --- | --- | --- | --- | --- | --- | --- |
| Keystroke | t-value | p-value | t-value | p-value | t-value | p-value |
| E-2 | 1.57 | 0.13 | **-3.01** | **0.007** | **3.81** | **0.001** |
| E-1 | **-10.15** | **< 0.001** | -2.63 | 0.016 | **-7.69** | **< 0.001** |
| E | **-13.56** | **< 0.001** | **-3.40** | **0.003** | **-5.77** | **< 0.001** |
| E+3 | **-4.27** | **< 0.001** | **-4.57** | **< 0.001** | 2.36 | 0.028 |

*Note:* Bold values indicate significant effects (Bonferroni corrected to *p* < 0.0125).

Self keystroke velocity during other-produced error and correct sequences (i.e., other velocity) was analysed with a 2 x 2 x 7 ANOVA (Error/Correct x Agency x Keystroke). These analyses yielded no significant main effects or interactions (see Figure SM4b and Table SM11), thus no additional analyses were conducted.

Table SM11
*ANOVA values for behavioural analysis of keystroke velocity during performance of error and correct sequences by partner with factors of Error/Correct x Agency x Keystroke.*

| Effect | *df* | *F* | *p*-value | *η_p_^2^* |
| --- | --- | --- | --- | --- |
| E/C | (1, 26) | 0.08 | 0.787 | 0.003 |
| Agency | (1, 26) | 1.86 | 0.184 | 0.067 |
| Keystroke | (4.77, 124.09) | 2.30 | 0.051 | 0.081 |
| E/C x Agency | (1, 26) | 0.22 | 0.643 | 0.008 |
| E/C x Keystroke | (4.82, 125.34) | 1.91 | 0.1 | 0.068 |
| Agency x Keystroke | (4.92, 127.84) | 0.83 | 0.527 | 0.031 |
| E/C x Agency x Keystroke | (4.92, 127.97) | 0.83 | 0.532 | 0.031 |

*Note:* E/C = Error/Correct.

**SM3: EEG Results**

**SM3A: *Error-related Negativity (ERN)***

Self errors elicited the ERN component at a latency of 30 – 90 ms (see Figure SM6). A 2 x 2 x 3 x 3 (Error/Correct x Agency x Lateralisation [left/middle/right] x Anterior/Posterior [anterior/centre/posterior]) ANOVA on data in the 30-90 ms time window yielded an interaction of Error/Correct and Anterior/Posterior (*F*(1.12, 39.21) = 6.55, *p* = 0.012) and a 3-way interaction between Agency, Lateralisation, and Anterior/Posterior, *F*(3.08, 107.95) = 2.71, *p* = 0.047. No other main effects or interactions were significant. Full ANOVA results are shown in Table SM12. After breaking the ANOVA down by Anterior/Posterior (anterior/centre/posterior), a main effect of Error/Correct was found in the Anterior (*F*(1, 35) = 2.28, *p* = 0.029) region of interest (see Figures SM6 and SM7). There were no other main effects or interactions. Full ANOVA results are shown in Table SM13. The amplitude of the ERN when playing an error was significantly larger than when playing a correct keystroke in anterior regions of interest, regardless of Agency or Lateralisation – *t*(35) = 2.28, *p* = 0.029 (see Figures SM6 and SM7).


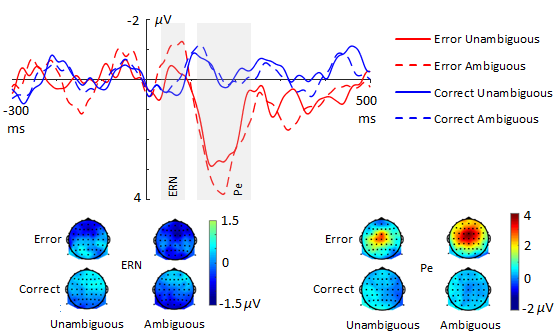


Figure SM6.Grand-averaged waveforms showing the ERN (30 – 90 ms) and Pe (120 – 230 ms) during self performance time-locked to onset of correct (blue) and error (red) keystrokes at electrode FCz. The solid lines represent playing in the unambiguous condition, dashed lines represent playing in the ambiguous condition. Shown below are scalp voltage distributions for each condition for the ERN (at 60 ms; left) and the Pe (at 170 ms; right).


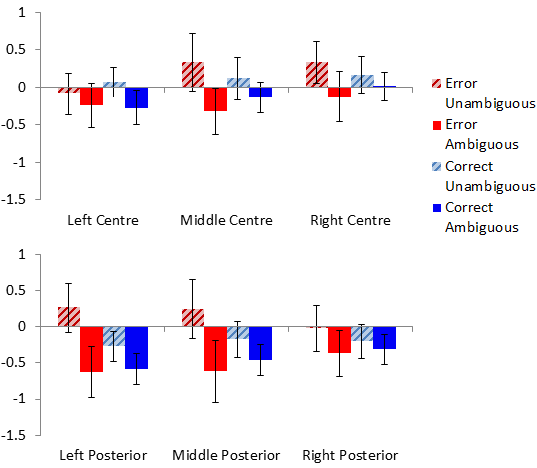

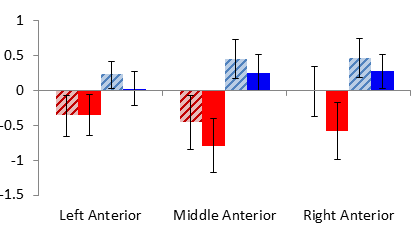

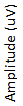

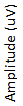

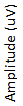


*

Figure SM7. Amplitude (in microvolts) of ERN for regions of interest time-locked to error and correct keystrokes in ambiguous and unambiguous conditions of agency.

Table SM12
*ANOVA values for analysis of ERN during performance of error and correct keystrokes at a time window of 30 – 90 ms post-error onset with factors of Error/Correct x Agency x Lateralization x Anterior/Posterior.*

| Effect | *df* | *F* | *p*-value | *η_p_^2^* |
| --- | --- | --- | --- | --- |
| E/C | (1, 35) | 0.68 | 0.414 | 0.019 |
| Agency | (1, 35) | 2.18 | 0.149 | 0.059 |
| L/R | (1.98, 69.28) | 1.72 | 0.186 | 0.047 |
| A/P | (1.18, 41.43) | 2.89 | 0.091 | 0.076 |
| E/C x Agency | (1, 35) | 0.30 | 0.589 | 0.008 |
| E/C x L/R | (1.53, 53.56) | 0.40 | 0.617 | 0.011 |
| Agency x L/R | (1.66, 57.98) | 0.43 | 0.615 | 0.012 |
| E/C x Agency x L/R | (1.82, 63.78) | 0.60 | 0.538 | 0.017 |
| **E/C x A/P** | **(1.12, 39.21)** | **6.55** | **0.012** | **0.158** |
| Agency x A/P | (1.11, 38.97) | 0.45 | 0.529 | 0.013 |
| E/C x Agency x A/P | (1.89, 38.11) | 0.46 | 0.517 | 0.013 |
| L/R x A/P | (2.86, 99.93) | 1.12 | 0.344 | 0.031 |
| E/C x L/R x A/P | (2.48, 86.94) | 1.98 | 0.134 | 0.053 |
| **Agency x L/R x A/P** | **(3.08, 107.95)** | **2.71** | **0.047** | **0.072** |
| E/C x Agency x L/R x A/P | (3.26, 114.05) | 1.99 | 0.114 | 0.054 |

*Note:* Bold values indicate significant effects (*p* < 0.05). E/C = Error/Correct; L/R = Left/Middle/Right; A/P = Anterior/Centre/Posterior.

Table SM13
*ANOVA values for analysis of ERN during performance of error and correct keystrokes at a time window of 30 – 90 ms post-error onset with factors of Error/Correct x Agency x Lateralization for Anterior regions of interest.*

| Effect | *df* | *F* | *p*-value | *η_p_^2^* |
| --- | --- | --- | --- | --- |
| **E/C** | **(1, 35)** | **5.20** | **0.029** | **0.129** |
| Agency | (1, 35) | 0.72 | 0.402 | 0.02 |
| L/R | (1.97, 68.86) | 2.47 | 0.093 | 0.066 |
| E/C x Agency | (1, 35) | 0.04 | 0.84 | 0.001 |
| E/C x L/R | (1.58, 55.33) | 1.89 | 0.169 | 0.051 |
| Agency x L/R | (1.61, 56.28) | 1.22 | 0.295 | 0.034 |
| E/C x Agency x L/R | (1.78, 62.31) | 1.44 | 0.246 | 0.039 |

*Note:* Bold values indicate significant effects (*p* < 0.05). E/C = Error/Correct; L/R = Left/Middle/Right.

In addition, we conducted additional ANOVAs to increase signal-to-noise ratio for detecting effects of Agency: one to identify ROIs in which EEG activity differed between error and correct responses regardless of Agency and follow-up ANOVAs that included only self-produced error responses (i.e., without correct responses) separated between Agency conditions in the relevant ROIs. These additional analyses revealed no such effects (see Table SM14).

Table SM14
*Statistical values for ANOVAs and follow-up analyses of ERN during performance of error and correct keystrokes at a time window of 30 to 90 ms post-error onset with factors of Error/Correct x Lateralization x Anterior/Posterior. Follow-up tests include Agency in ROIs where a difference was found between Error and Correct.*

| Effect (ANOVA) | *df* | *F* | *p*-value | *η_p_^2^* |
| --- | --- | --- | --- | --- |
| E/C | (1, 35) | 0.63 | 0.434 | 0.018 |
| L/R | (1.94, 67.82) | 1.92 | 0.156 | 0.052 |
| **A/P** | **(1.24, 43.21)** | **4.17** | **0.039** | **0.106** |
| E/C x L/R | (1.48, 51.73) | 0.39 | 0.617 | 0.011 |
| **E/C x A/P** | **(1.14, 40.04)** | **6.52** | **0.012** | **0.157** |
| L/R x A/P | (2.75, 96.22) | 1.01 | 0.387 | 0.028 |
| E/C x L/R x A/P | (2.38, 83.45) | 1.78 | 0.168 | 0.048 |
| Effect (*t*-tests) | *df* | *t* | *p*-value |  |
| **Anterior: Correct vs Error** | **(1, 35)** | **2.28** | **0.029** |  |
| Centre: Correct vs Error | (1, 35) | 0.01 | 0.996 |  |
| Posterior: Correct vs Error | (1, 35) | -0.38 | 0.704 |  |
| *t*-test (Anterior ROIs) | *df* | *t* | *p*-value |  |
| Ambiguous vs Unambiguous | (1, 35) | 0.62 | 0.538 |  |

*Note:* Bold values indicate significant effects (*p* < 0.05). E/C = Error/Correct; L/R = Left/Middle/Right; A/P = Anterior/Centre/Posterior.

**SM3B: *Pre-ERN – Extra Note Errors and Wrong Note Errors***

*Lateralisation Split ANOVA Results*

See Table SM15 for full ANOVA results and Figure SM8 for amplitude graphs. Follow-up tests showed that in the Left Posterior region of interest, amplitude of the pre-ERN was more negative when playing wrong note errors (*t*(26) = 2.75, *p* = 0.011) and when playing correct notes (*t*(26) = 3.64, *p* = 0.001) than when playing extra note errors, but no significant difference was found between wrong note errors and correct notes (*t*(26) = 0.94, *p* = 0.335). In the Middle Posterior region of interest, amplitude of the pre-ERN was more negative when playing wrong note errors (*t*(26) = 3.57, *p* = 0.001) and when playing correct notes (*t*(26) = 2.38, *p* = 0.025) than when playing extra note errors, but no significant difference was found between wrong note errors and correct notes (*t*(26) = 1.92, *p* = 0.066). Finally, in the Right Centre region of interest, amplitude of the pre-ERN when playing wrong note errors was more negative than when playing extra note errors (*t*(26) = 2.68, *p* = 0.013) or when playing correct notes (*t*(26) = 2.28, *p* = 0.031), but there was no significant difference found between extra note errors and correct notes (*t*(26) = 0.90, *p* = 0.378). In the Right Posterior region of interest, amplitude of the pre-ERN when playing wrong note errors was more negative than when playing extra note errors (*t*(26) = 3.75, *p* = 0.001) or when playing correct notes (*t*(26) = 2.98, *p* = 0.006), but no significant difference was found between extra note errors and correct notes (*t*(26) = 1.62, *p* = 0.117).

Table SM15
*ANOVA values for analysis of pre-ERN during performance of error and correct keystrokes at a time window of 80 – 25 ms pre-error onset with factors of Extra/Wrong/Correct x Anterior/Posterior for Lateralised regions of interest.*

| Effect | | *df* | *F* | *p*-value | *η_p_^2^* |
| --- | --- | --- | --- | --- | --- |
| Left | E/W/C | (1.42, 37.01) | 3.51 | 0.055 | 0.119 |
|  | A/P | (1.22, 31.68) | 2.80 | 0.097 | 0.097 |
|  | **E/W/C x A/P** | **(1.86, 48.47)** | **4.06** | **0.026** | **0.135** |
| Middle | E/W/C | (1.64, 42.72) | 3.31 | 0.055 | 0.113 |
|  | A/P | (1.21, 31.52) | 1.36 | 0.26 | 0.05 |
|  | **E/W/C x A/P** | **(2.12, 55.03)** | **5.73** | **0.005** | **0.181** |
| Right | **E/W/C** | **(1.71, 44.56)** | **5.53** | **0.01** | **0.175** |
|  | A/P | (1.22, 31.82) | 0.27 | 0.653 | 0.01 |
|  | **E/W/C x A/P** | **(2.1, 54.63)** | **5.76** | **0.005** | **0.181** |

*Note:* Bold values indicate significant effects (*p* < 0.05). E/W/C = Extra note error/Wrong note error/Correct; A/P = Anterior/Centre/Posterior.

* = *p* < 0.05 between Extra and Wrong and Extra and Correct

** = *p* < 0.05 between Wrong and Extra and Wrong and Correct


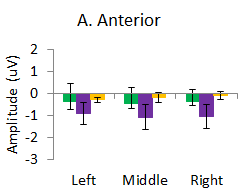

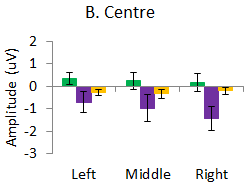

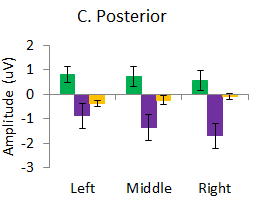

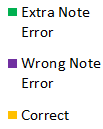


*

*

**

**

Figure SM8. Amplitude (in microvolts) of pre-ERN for regions of interest time-locked to extra note errors, wrong note errors, and correct notes.

**SM3C: *ERN – Extra Note Errors and Wrong Note Errors***

*Anterior/Posterior Split ANOVA Results*

See Table SM16 for full ANOVA results. Follow-up tests showed that the amplitude of the ERN more negative when playing extra note errors than when playing wrong note errors (*t*(26) = 2.86, *p* = 0.008) or when playing correct notes (*t*(26) = 2.26, *p* = 0.003) in the Middle Anterior region of interest only, but no significant difference between wrong note errors and correct notes (*t*(26) = 1.48, *p* = 0.151; see Figure SM9).

* = *p* < 0.05 between Extra and Wrong and Extra and Correct

*


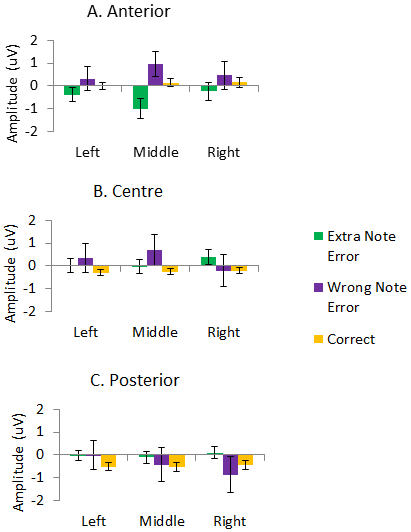


Figure SM9. Amplitude (in microvolts) of ERN for regions of interest time-locked to extra note errors, wrong note errors, and correct notes.

Table SM16
*ANOVA values for analysis of ERN during performance of error and correct keystrokes at a time window of 30 – 90 ms post-error onset with factors of Extra/Wrong/Correct x Lateralization for Anterior, Centre, and Posterior regions of interest.*

| Effect | | *df* | *F* | *p*-value | *η_p_^2^* |
| --- | --- | --- | --- | --- | --- |
| Anterior | E/W/C | (1.73, 45.07) | 2.35 | 0.114 | 0.083 |
|  | L/R | (1.95, 50.81) | 0.84 | 0.437 | 0.031 |
|  | **E/W/C x L/R** | **(2.53, 65.74)** | **3.03** | **0.044** | **0.104** |
| Centre | E/W/C | (1.33, 34.66) | 0.48 | 0.545 | 0.018 |
|  | L/R | (1.67, 43.46) | 0.54 | 0.554 | 0.02 |
|  | **E/W/C x L/R** | **(2.73, 70.95)** | **2.95** | **0.043** | **0.102** |
| Posterior | E/W/C | (1.27, 33.01) | 0.39 | 0.586 | 0.015 |
|  | L/R | (1.43, 37.16) | 0.84 | 0.406 | 0.031 |
|  | E/W/C x L/R | (2.1, 54.51) | 1.58 | 0.215 | 0.057 |

*Note:* Bold values indicate significant effects (*p* < 0.05). E/W/C = Extra note error/Wrong note error/Correct; L/R = Left/Middle/Right.

**SM3D: *Error Positivity (Pe)***

Based on previous research in error processing (Gehring, Liu, Orr, & Carp, 2012; Overbeek, Nieuwenhuis, & Ridderinkhof, 2005), a Pe was predicted to follow the ERN in the self EEG data. In the current study, a Pe was observed in the time window of 120 – 230 ms (see Figure SM6). A 2 x 2 x 3 x 3 (Error/Correct x Agency x Lateralisation [left/middle/right] x Anterior/Posterior [anterior/centre/posterior]) ANOVA was performed on the Pe. Full ANOVA results are shown in Table SM17. This analysis yielded a main effect of Error/Correct (*F*(1, 35) = 17.44, *p* < 0.001), a main effect of Lateralisation (*F*(1.99, 69.48) = 13.39, *p* < 0.001), a main effect of Anterior/Posterior (*F*(1.31, 45.99) = 5.96, *p* = 0.012, an interaction of Error/Correct and Lateralisation (*F*(1.92, 67.35) = 12.41, *p* < 0.001), an interaction of Error/Correct and Anterior/Posterior (*F*(1.31, 45.80) = 8.56, *p* = 0.003), an interaction of Lateralisation and Anterior/Posterior (*F*(3.16, 110.44) = 5.51, *p* = 0.001), and a 3-way interaction between Error/Correct, Lateralisation, and Anterior/Posterior (*F*(3.36, 117.74) = 5.32, *p* = 0.001).

Table SM17
*ANOVA values for analysis of Pe during performance of error and correct keystrokes at a time window of 120 – 230 ms post-error onset with factors of Error/Correct x Agency x Lateralization x Anterior/Posterior.*

| Effect | *df* | *F* | | *p*-value | *η_p_^2^* |
| --- | --- | --- | --- | --- | --- |
| **E/C** | **(1, 35)** | | **17.44** | **< 0.001** | **0.333** |
| Agency | (1, 35) | | 1.62 | 0.212 | 0.044 |
| **L/R** | **(1.99, 69.48)** | | **13.39** | **< 0.001** | **0.277** |
| **A/P** | **(1.31, 46)** | | **5.96** | **0.012** | **0.146** |
| E/C x Agency | (1, 35) | | 0.31 | 0.582 | 0.009 |
| **E/C x L/R** | **(1.92, 67.35)** | | **12.41** | **< 0.001** | **0.262** |
| Agency x L/R | (1.65, 57.71) | | 0.55 | 0.547 | 0.015 |
| E/C x Agency x L/R | (1.89, 66.08) | | 0.92 | 0.398 | 0.026 |
| **E/C x A/P** | **(1.31, 45.80)** | | **8.56** | **0.003** | **0.196** |
| Agency x A/P | (1.08, 37.81) | | 2.01 | 0.164 | 0.054 |
| E/C x Agency x A/P | (1.10, 38.57) | | 1.80 | 0.188 | 0.049 |
| **L/R x A/P** | **(3.16, 110.44)** | | **5.51** | **0.001** | **0.136** |
| **E/C x L/R x A/P** | **(3.36, 117.74)** | | **5.32** | **0.001** | **0.132** |
| Agency x L/R x A/P | (3.02, 105.76) | | 1.70 | 0.172 | 0.046 |
| E/C x Agency x L/R x A/P | (2.77, 96.84) | | 0.59 | 0.613 | 0.016 |

*Note:* Bold values indicate significant effects (*p* < 0.05). E/C = Error/Correct; L/R = Left/Middle/Right; A/P = Anterior/Centre/Posterior.

Breaking the ANOVA down by Anterior/Posterior showed effects at each level of Anterior, Centre, and Posterior regions of interest (see Figure SM10). In the Anterior regions of interest, the analysis revealed a main effect of Error/Correct (*F*(1, 35) = 18.48, *p* < 0.001), a main effect of Lateralisation (*F*(1.89, 65.40) = 9.84, *p* < 0.001), and an interaction between Error/Correct and Lateralisation (*F*(2, 69.26) = 7.51, *p* = 0.001). Full ANOVA results are shown in Table SM18. Amplitude of the Pe was larger when playing error keystrokes than when playing correct keystrokes, regardless of Agency or Lateralisation (*t*(35) = 4.30, *p* < 0.001; see Figures SM6 and SM10).


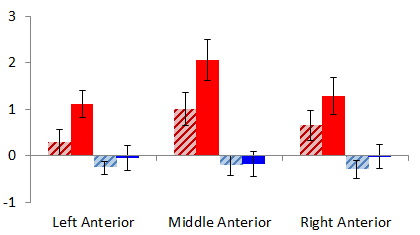

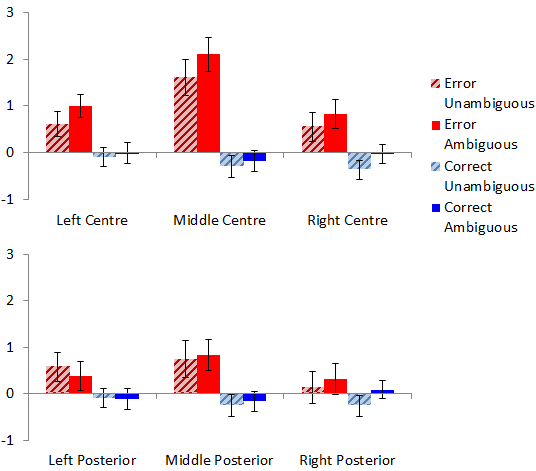

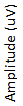

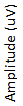

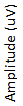


*

*

*

* = *p* < 0.05 between Error and Correct, regardless of Agency

*

*

*

*

*

Figure SM10. Amplitude (in microvolts) of Pe for regions of interest time-locked to error and correct keystrokes in ambiguous and unambiguous conditions of agency.

Table SM18
*ANOVA values for analysis of Pe during performance of error and correct keystrokes at a time window of 120 – 230 ms with factors of Error/Correct x Agency x Lateralization for Anterior regions of interest.*

| Effect | *df* | *F* | *p*-value | *η_p_^2^* |
| --- | --- | --- | --- | --- |
| **E/C** | **(1, 35)** | **18.48** | **< 0.001** | **0.346** |
| Agency | (1, 35) | 3.07 | 0.089 | 0.081 |
| **L/R** | **(1.89, 65.40)** | **9.84** | **< 0.001** | **0.219** |
| E/C x Agency | (1, 35) | 1.92 | 0.175 | 0.052 |
| **E/C x L/R** | **(2, 69.26)** | **7.51** | **0.001** | **0.177** |
| Agency x L/R | (1.84 64.49) | 0.16 | 0.838 | 0.004 |
| E/C x Agency x L/R | (1.61, 56.25) | 1.50 | 0.234 | 0.041 |

*Note:* Bold values indicate significant effects (*p* < 0.05). E/C = Error/Correct; L/R = Left/Middle/Right.

In the Centre regions of interest, there was a main effect of Error/Correct (*F*(1, 35) = 22.80, *p* < 0.001), a main effect of Lateralisation (*F*(1.98, 69.45) = 17.82, *p* < 0.001), and an interaction between Error/Correct and Lateralisation (*F*(1.89, 65.99) = 16.90, *p* < 0.001). Full ANOVA results are shown in Table SM19. Amplitude of the Pe was larger when playing error keystrokes than when playing correct keystrokes, regardless of Agency or Lateralisation (*t*(35) = 4.78, *p* < 0.001; see Figures SM6 and SM10).

Table SM19
*ANOVA values for analysis of Pe during performance of error and correct keystrokes at a time window of 120 – 230 ms with factors of Error/Correct x Agency x Lateralization for Centre regions of interest.*

| Effect | *df* | *F* | *p*-value | *η_p_^2^* |
| --- | --- | --- | --- | --- |
| **E/C** | **(1, 35)** | **22.80** | **< 0.001** | **0.394** |
| Agency | (1, 35) | 1.56 | 0.22 | 0.043 |
| **L/R** | **(1.98, 69.45)** | **17.82** | **< 0.001** | **0.337** |
| E/C x Agency | (1, 35) | 0.22 | 0.642 | 0.006 |
| **E/C x L/R** | **(1.89, 65.99)** | **16.90** | **< 0.001** | **0.326** |
| Agency x L/R | (1.77, 61.99) | 0.13 | 0.855 | 0.004 |
| E/C x Agency x L/R | (1.74, 60.88) | 0.66 | 0.502 | 0.018 |

*Note:* Bold values indicate significant effects (*p* < 0.05). E/C = Error/Correct; L/R = Left/Middle/Right.

In the Posterior regions of interest, there was a main effect of Error/Correct (*F*(1, 35) = 6.147, *p* = 0.018) and an interaction between Error/Correct and Lateralisation (*F*(1.51, 52.67) = 5.38, *p* = 0.013). Full ANOVA results are shown in Table SM20. Amplitude of the Pe was larger when playing error keystrokes than when playing correct keystrokes in the left posterior (*t*(35) = 2.27, *p* = 0.029) and middle posterior (*t*(35) = 3.17, *p* = 0.003) regions of interest, regardless of Agency condition, but not in the right posterior region of interest (*t*(35) = 1.15, *p* = 0.256; see Figures SM6 and SM10).

Table SM20
*ANOVA values for analysis of Pe during performance of error and correct keystrokes at a time window of 120 – 230 ms with factors of Error/Correct x Agency x Lateralization for Posterior regions of interest.*

| Effect | *df* | *F* | *p*-value | *η_p_^2^* |
| --- | --- | --- | --- | --- |
| **E/C** | **(1, 35)** | **6.15** | **0.018** | **0.149** |
| Agency | (1, 35) | 0.10 | 0.749 | 0.003 |
| L/R | (1.88, 65.76) | 2.69 | 0.078 | 0.071 |
| E/C x Agency | (1, 35) | 0.03 | 0.854 | 0.001 |
| **E/C x L/R** | **(1.51, 52.67)** | **5.38** | **0.013** | **0.133** |
| Agency x L/R | (1.66, 58.12) | 2.54 | 0.097 | 0.068 |
| E/C x Agency x L/R | (1.93, 67.68) | 0.15 | 0.852 | 0.004 |

*Note:* Bold values indicate significant effects (*p* < 0.05). E/C = Error/Correct; L/R = Left/Middle/Right.

Supplementary ANOVAs to increase signal-to-noise ratio for detecting effects of Agency did not reveal effects of Agency (see Table SM21).

Table SM21
*Statistical values for ANOVAs and follow-up analyses of Pe during performance of error and correct keystrokes at a time window of 120 – 230 ms pre-error onset with factors of Error/Correct x Lateralisation x Anterior/Posterior. Follow-up tests include Agency in ROIs where a difference was found between Error and Correct.*

| Effect (ANOVA) | *df* | *F* | *p*-value | *η_p_^2^* |
| --- | --- | --- | --- | --- |
| **E/C** | **(1, 35)** | **17.04** | **< 0.001** | **0.327** |
| **L/R** | **(2, 69.87)** | **14.91** | **< 0.001** | **0.299** |
| **A/P** | **(1.31, 45.94)** | **8.33** | **0.003** | **0.192** |
| **E/C x L/R** | **(1.89, 66.20)** | **13.41** | **< 0.001** | **0.277** |
| **E/C x A/P** | **(1.36, 47.59)** | **15.75** | **< 0.001** | **0.31** |
| **L/R x A/P** | **(3.17, 111.04)** | **7.45** | **< 0.001** | **0.175** |
| **E/C x L/R x A/P** | **(3.26, 113.94)** | **5.8** | **0.001** | **0.142** |
| Effect (*t*-tests) | *df* | *t* | *p*-value |  |
| **Anterior: Correct vs Error** | **(1, 35)** | **-4.843** | **< 0.001** |  |
| **Centre: Correct vs Error** | **(1, 35)** | **-4.735** | **< 0.001** |  |
| **Posterior: Correct vs Error** | **(1, 35)** | **-2.081** | **0.045** |  |
| Effect (*t*-tests) | *df* | *t* | *p*-value |  |
| **Left: Correct vs Error** | **(1, 35)** | **-3.295** | **0.002** |  |
| **Middle: Correct vs Error** | **(1, 35)** | **-5.017** | **< 0.001** |  |
| **Right: Correct vs Error** | **(1, 35)** | **-2.908** | **0.006** |  |
| Effect (ANOVA in All ROIs) | *df* | *F* | *p*-value | *η_p_^2^* |
| **L/R** | **(2, 69.83)** | **20.636** | **< 0.001** | **0.371** |
| **A/P** | **(1.28, 44.76)** | **11.093** | **0.001** | **0.241** |
| Agency | (1, 35) | 0.84 | 0.366 | 0.023 |
| **L/R x A/P** | **(3.17, 110.78)** | **7.261** | **< 0.001** | **0.172** |
| L/R x Agency | (1.77, 61.78) | 0.731 | 0.47 | 0.02 |
| A/P x Agency | (1.07, 37.45) | 2.669 | 0.109 | 0.071 |
| L/R x A/P x Agency | (2.75, 96.12) | 1.052 | 0.369 | 0.029 |

*Note:* Bold values indicate significant effects (*p* < 0.05). E/C = Error/Correct; L/R = Left/Middle/Right; A/P = Anterior/Centre/Posterior.

**SM3E: *Pe – Extra Note Errors and Wrong Note Errors***

Anterior/Posterior Split ANOVA Results

Breaking the ANOVA down by Anterior/Posterior revealed significant effects in Anterior, Centre, and Posterior regions of interest. In the Anterior regions of interest, there was a significant main effect of Extra/Wrong/Correct (*F*(1.53, 39.88) = 3.94, *p* = 0.037) and a significant main effect of Lateralisation (*F*(1.92, 49.83) = 5.94, *p* = 0.005), but no significant interaction (see Table SM22). Amplitude of the Pe was greater when playing extra note errors than when playing correct notes (*t*(26) = 4.62, *p* < 0.001) in the Middle Anterior region of interest, but there were no significant differences between extra note errors and wrong note errors (*t*(26) = 1.44, *p* = 0.162) or between wrong note errors and correct notes (*t*(26) = 1.22, *p* = 0.223; see Figure SM11).


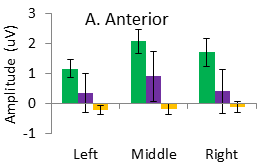

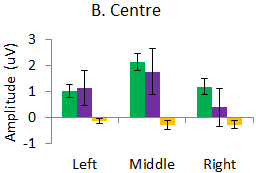

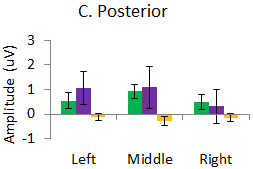

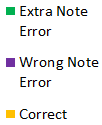


*

* = *p* < 0.05 between Extra and Correct

** = *p* < 0.05 between Extra and Correct and Wrong and Correct

*** = *p* < 0.05 between Middle and Right

**

***

Figure SM11. Amplitude (in microvolts) of Pe for regions of interest time-locked to extra note errors, wrong note errors, and correct notes.

Table SM22
*ANOVA values for analysis of Pe during performance of error and correct keystrokes at a time window of 120 – 230 ms pre-error onset with factors of Extra/Wrong/Correct x Lateralisation for Anterior, Centre, and Posterior regions of interest.*

| Effect | | *df* | *F* | *p*-value | *η_p_^2^* |
| --- | --- | --- | --- | --- | --- |
| Anterior | **E/W/C** | **(1.53, 39.88)** | **3.94** | **0.037** | **0.186** |
|  | **L/R** | **(1.92, 49.83)** | **5.94** | **0.005** | **0.132** |
|  | E/W/C x L/R | (2.24, 58.15) | 1.15 | 0.328 | 0.042 |
| Centre | **E/W/C** | **(1.27, 33.08)** | **3.86** | **0.049** | **0.129** |
|  | **L/R** | **(1.75, 45.51)** | **13.33** | **< 0.001** | **0.339** |
|  | **E/W/C x L/R** | **(2.59, 67.3)** | **3.64** | **0.022** | **0.123** |
| Posterior | E/W/C | (1.24, 32.25) | 1.33 | 0.267 | 0.048 |
|  | **L/R** | **(1.84, 47.75)** | **3.38** | **0.046** | **0.115** |
|  | E/W/C x L/R | (2.56, 66.6) | 1.85 | 0.154 | 0.067 |

*Note:* Bold values indicate significant effects (*p* < 0.05). E/W/C = Extra note error/Wrong note error/Correct; L/R = Left/Middle/Right.

In the Centre regions of interest, there was a significant main effect of Extra/Wrong/Correct (*F*(1.27, 33.08) = 3.86, *p* = 0.049), a significant main effect of Lateralisation (*F*(1.75, 45.51) = 13.33, *p* < 0.001), and a significant interaction between Lateralisation and Extra/Wrong/Correct (*F*(2.59, 67.30) = 3.64, *p* = 0.022; see Table SM22). Amplitude of the Pe was greater when playing extra note errors than when playing correct notes (*t*(26) = 5.69, *p* < 0.001) and when playing wrong note errors than when playing correct notes (*t*(26) = 2.12, *p* = 0.044) in the Middle Centre region of interest, but there was no significant difference between extra note errors and wrong note errors (*t*(26) = 0.44, *p* = 0.664; see Figure SM11).

In the Posterior regions of interest, there was a significant main effect of Lateralisation (*F*(1.84, 47.75) = 3.38, *p* = 0.046), but no significant main effect of Extra/Wrong/Correct or significant interaction between Lateralisation and Extra/Wrong/Correct (see Table SM22). Further analysis showed the amplitude of the Pe was greater in the Middle Posterior region of interest than the Right Posterior region of interest, regardless of error type (*t*(26) = 2.911, *p* = 0.007; see Figure SM11).

**SM3F: *Feedback-related Negativity (FRN)***

The FRN elicited by errors performed by a partner was observed between 215 to 300 ms, with a more parietal distribution than the ERN or Pe (see Figure SM12). The 2 x 2 x 3 x 3 (Error/Correct x Agency x Lateralisation [left/middle/right] x Anterior/Posterior [anterior/centre/posterior]) ANOVA on this component yielded a main effect of Error (*F*(1, 35) = 5.91, *p* = 0.02) and a main effect of Anterior/Posterior (*F*(1.20, 42.03) = 5.72, *p* = 0.016). Full ANOVA results are shown in Table SM23.


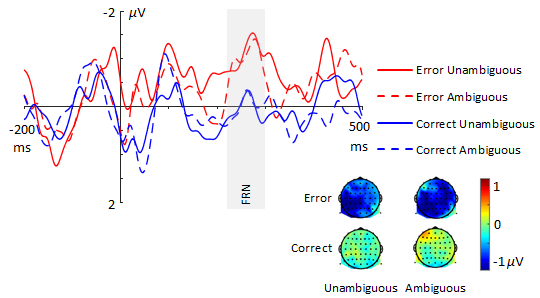


Figure SM12. Grand-averaged waveforms showing FRN (215 – 300 ms, shaded) during self performance time-locked to onset of other-produced correct (blue) and error (red) notes during unambiguous (solid) and ambiguous (dashed) trials at electrode CPz. Shown below are scalp voltage distributions for each condition at 275 ms.

Table SM23 *ANOVA values for analysis of FRN during performance of error and correct keystrokes at a time window of 215 – 300 ms with factors of Error/Correct x Agency x Lateralization x Anterior/Posterior.*

| Effect | *df* | *F* | *p*-value | *η_p_^2^* |
| --- | --- | --- | --- | --- |
| **E/C** | **(1, 35)** | **5.91** | **0.02** | **0.144** |
| Agency | (1, 35) | 0.17 | 0.684 | 0.005 |
| L/R | (1.80, 63.04) | 1.08 | 0.339 | 0.03 |
| **A/P** | **(1.20, 42.03)** | **5.72** | **0.016** | **0.14** |
| E/C x Agency | (1, 35) | 0.08 | 0.775 | 0.002 |
| E/C x L/R | (1.82, 63.58) | 2.51 | 0.095 | 0.067 |
| Agency x L/R | (1.62, 56.69) | 0.53 | 0.555 | 0.015 |
| E/C x Agency x L/R | (1.65, 57.88) | 0.66 | 0.496 | 0.018 |
| E/C x A/P | (1.24, 43.22) | 0.98 | 0.347 | 0.027 |
| Agency x A/P | (1.29, 44.99) | 0.59 | 0.486 | 0.017 |
| E/C x Agency x A/P | (1.32, 46.14) | 0.58 | 0.495 | 0.016 |
| L/R x A/P | (3.11, 108.69) | 2.47 | 0.064 | 0.066 |
| E/C x L/R x A/P | (2.86, 100.24) | 0.91 | 0.434 | 0.025 |
| Agency x L/R x A/P | (2.43, 85.05) | 1.76 | 0.17 | 0.048 |
| E/C x Agency x L/R x A/P | (2.97, 103.96) | 0.75 | 0.524 | 0.021 |

*Note:* Bold values indicate significant effects (*p* < 0.05). E/C = Error/Correct; L/R = Left/Middle/Right; A/P = Anterior/Centre/Posterior.

Breaking the ANOVA down by Anterior/Posterior revealed significant effects in the Centre and Posterior ROIs (see Figures SM12 and SM13). The Centre ROIs revealed a significant main effect of Error/Correct. Full ANOVA results are shown in Table SM24. The amplitude of the FRN was more negative in response to the partner’s error keystrokes than for the partner’s correct keystrokes, regardless of Agency or Lateralisation (*t*(35) = 2.64, *p* = 0.012).

*

*

*

*

*


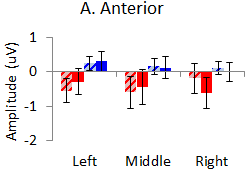

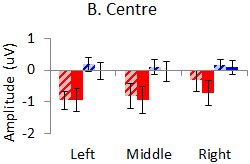

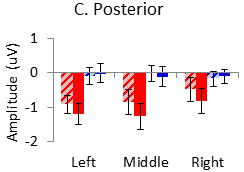

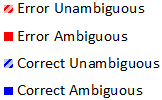


* = *p* < 0.05 between Error and Correct, regardless of Agency

Figure SM13. Amplitude (in microvolts) of FRN for regions of interest time-locked to error and correct keystrokes in ambiguous and unambiguous conditions of agency.

Table SM24
*ANOVA values for analysis of FRN during performance of error and correct keystrokes at a time window of 215 to 300 ms with factors of Error/Correct x Agency x Lateralization for Centre regions of interest.*

| Effect | *df* | *F* | *p*-value | *η_p_^2^* |
| --- | --- | --- | --- | --- |
| **E/C** | **(1, 35)** | **6.99** | **0.012** | **0.166** |
| Agency | (1, 35) | 0.30 | 0.588 | 0.008 |
| L/R | (1.90, 66.33) | 2.23 | 0.118 | 0.060 |
| E/C x Agency | (1, 35) | 0.004 | 0.948 | 0.000 |
| E/C x L/R | (1.77, 61.99) | 1.52 | 0.227 | 0.042 |
| Agency x L/R | (1.75, 61.13) | 0.23 | 0.763 | 0.007 |
| E/C x Agency x L/R | (1.48, 51.61) | 0.97 | 0.363 | 0.027 |

*Note:* Bold values indicate significant effects (*p* < 0.05). E/C = Error/Correct; L/R = Left/Middle/Right.

The Posterior ROIs revealed a main effect of Error/Correct and an interaction between Error/Correct and Lateralisation. Full ANOVA results are shown in Table SM25. The FRN was significantly more negative when playing error keystrokes than when playing correct keystrokes in the Left Posterior (*t*(35) = 3.35, *p* = 0.002) and Middle Posterior (*t*(35) = 2.69, *p* = 0.011) ROIs, regardless of Agency condition, but not in the Right Posterior ROI (*t*(35) = 1.60, *p* = 0.118; see Figure SM13).

Table SM25
*ANOVA values for analysis of FRN during performance of error and correct keystrokes at a time window of 215 to 300 ms with factors of Error/Correct x Agency x Lateralization for Posterior regions of interest.*

| Effect | *df* | *F* | *p*-value | *η_p_^2^* |
| --- | --- | --- | --- | --- |
| **E/C** | **(1, 35)** | **7.11** | **0.012** | **0.169** |
| Agency | (1, 35) | 0.36 | 0.552 | 0.010 |
| L/R | (1.84, 64.25) | 1.68 | 0.196 | 0.046 |
| E/C x Agency | (1, 35) | 0.68 | 0.416 | 0.019 |
| **E/C x L/R** | **(1.96, 68.70)** | **4.88** | **0.011** | **0.122** |
| Agency x L/R | (1.79, 62.61) | 0.31 | 0.708 | 0.009 |
| E/C x Agency x L/R | (1.79, 62.59) | 0.02 | 0.969 | 0.001 |

*Note:* Bold values indicate significant effects (*p* < 0.05). E/C = Error/Correct; L/R = Left/Middle/Right.

A supplementary ANOVA that included only self-produced error responses did not reveal effects of Agency (see Table SM26).

Table SM26
*Statistical values for ANOVAs and follow-up analyses of FRN during performance of error and correct keystrokes at a time window of 215 – 300 ms pre-error onset with factors of Error/Correct x Lateralisation x Anterior/Posterior. Follow-up tests include Agency in ROIs where a difference was found between Error and Correct.*

| Effect (ANOVA) | *df* | *F* | *p*-value | *η_p_^2^* |
| --- | --- | --- | --- | --- |
| **E/C** | **(1, 35)** | **19.68** | **< 0.001** | **0.36** |
| L/R | (1.93, 67.38) | 1.86 | 0.163 | 0.051 |
| **A/P** | **(1.18, 41.35)** | **11.53** | **0.001** | **0.248** |
| **E/C x L/R** | **(1.70, 59.56)** | **4.79** | **0.016** | **0.12** |
| E/C x A/P | (1.24, 43.35) | 0.43 | 0.56 | 0.012 |
| L/R x A/P | (3.18, 111.13) | 1.54 | 0.207 | 0.042 |
| E/C x L/R x A/P | (2.88, 100.61) | 0.89 | 0.447 | 0.025 |
| Effect (*t*-tests) | *df* | *t* | *p*-value |  |
| **Anterior: Correct vs Error** | **(1, 35)** | **3.71** | **0.001** |  |
| **Centre: Correct vs Error** | **(1, 35)** | **4.74** | **< 0.001** |  |
| **Posterior: Correct vs Error** | **(1, 35)** | **3.94** | **< 0.001** |  |
| Effect (*t*-tests) | *df* | *t* | *p*-value |  |
| **Left: Correct vs Error** | **(1, 35)** | **5.08** | **< 0.001** |  |
| **Middle: Correct vs Error** | **(1, 35)** | **4.33** | **< 0.001** |  |
| **Right: Correct vs Error** | **(1, 35)** | **3.09** | **0.004** |  |
| Effect (ANOVA in All ROIs) | *df* | *F* | *p*-value | *η_p_^2^* |
| **L/R** | (1.91, 66.78) | 2.56 | 0.088 | 0.068 |
| **A/P** | **(1.18, 41.20)** | **3.95** | **0.047** | **0.101** |
| Agency | (1, 35) | 0.18 | 0.674 | 0.005 |
| **L/R x A/P** | (2.75, 96.25) | 1.91 | 0.138 | 0.052 |
| L/R x Agency | (1.55, 54.27) | 0.77 | 0.439 | 0.021 |
| A/P x Agency | (1.27, 44.30) | 0.68 | 0.449 | 0.019 |
| L/R x A/P x Agency | (2.72, 95.04) | 1.60 | 0.2 | 0.044 |

*Note:* Bold values indicate significant effects (*p* < 0.05). E/C = Error/Correct; L/R = Left/Middle/Right; A/P = Anterior/Centre/Posterior.

**SM3G: *FRN – Extra Note Errors and Wrong Note Errors***

*Anterior/Posterior Split ANOVA Results*

Breaking the ANOVA down by Anterior/Posterior revealed significant effects in Anterior, Centre, and Posterior ROIs. In the Anterior ROIs, there was a significant main effect of Extra/Wrong/Correct (*F*(1.55, 38.77) = 6.39, *p* = 0.007), a significant main effect of Lateralisation (*F*(1.97, 49.16) = 3.26, *p* = 0.048), and a significant interaction between Extra/Wrong/Correct and Lateralisation (*F*(2.53, 63.16) = 3.58, *p* = 0.025; see Table SM27). Amplitude of the FRN was greater when the partner played extra note errors than when the partner played wrong note errors in the Left (*t*(25) = 2.12, *p* = 0.044), Middle (*t*(25) = 3.15, *p* = 0.004), and Right (*t*(25) = 2.42, *p* = 0.023) Anterior ROIs. Amplitude of the FRN was also greater when the partner played correct notes than when the partner played wrong note errors in the Middle (*t*(25) = 3.06, *p* = 0.005) and Right (*t*(25) = 2.51, *p* = 0.019) Anterior regions of interest, but not in the Left Anterior ROI (*t*(25) = 2.05, *p* = 0.051). There were no significant differences between extra note errors and correct notes (all *p*-values > 0.25; see Figure SM14).


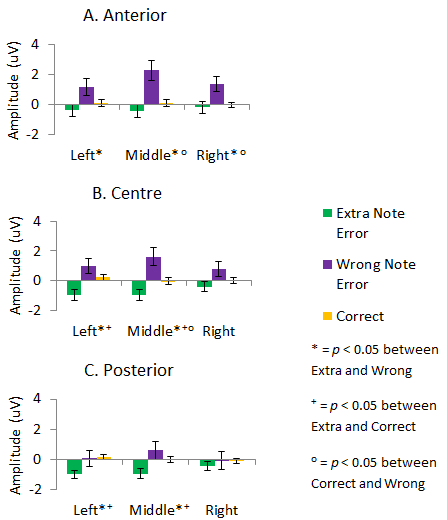


Figure SM14. Amplitude (in microvolts) of FRN for regions of interest time-locked to extra note errors, wrong note errors, and correct notes.

Table SM27
*ANOVA values for analysis of FRN during performance of error and correct keystrokes at a time window of 250 – 340 ms pre-error onset with factors of Extra/Wrong/Correct x Lateralisation for Anterior, Centre, and Posterior regions of interest.*

| Effect | | *df* | *F* | *p*-value | *η_p_^2^* |
| --- | --- | --- | --- | --- | --- |
| Anterior | **E/W/C** | **(1.55, 38.77)** | **6.39** | **0.007** | **0.204** |
|  | **L/R** | **(1.97, 49.16)** | **3.26** | **0.048** | **0.115** |
|  | **E/W/C x L/R** | **(2.53, 63.16)** | **3.58** | **0.025** | **0.125** |
| Centre | **E/W/C** | **(1.85, 46.26)** | **9.14** | **0.001** | **0.268** |
|  | L/R | (1.67, 41.66) | 0.261 | 0.731 | 0.01 |
|  | **E/W/C x L/R** | **(2.39, 59.82)** | **3.27** | **0.037** | **0.116** |
| Posterior | E/W/C | (1.47, 36.82) | 2.54 | 0.107 | 0.015 |
|  | L/R | (1.67, 41.69) | 0.37 | 0.653 | 0.015 |
|  | **E/W/C x L/R** | **(2.08, 51.87)** | **4.30** | **0.018** | **0.147** |

*Note:* Bold values indicate significant effects (*p* < 0.05). E/W/C = Extra note error/Wrong note error/Correct; L/R = Left/Middle/Right.

In the Centre regions of interest, there was a significant main effect of Extra/Wrong/Correct (*F*(1.85, 46.26) = 9.14, *p* = 0.001) and a significant interaction between Extra/Wrong/Correct and Lateralisation (*F*(2.39, 59.82) = 3.27, *p* = 0.037; see Table SM27). Amplitude of the FRN was greater when the partner played extra note errors than when the partner played wrong note errors in the Left (*t*(25) = 3.40, *p* = 0.002) and Middle (*t*(25) = 4.15, *p* < 0.001) Anterior regions of interest. Amplitude of the FRN was also greater when the partner played extra note errors than when the partner played correct notes in the Left (*t*(25) = 3.01, *p* = 0.006) and Middle (*t*(25) = 2.26, *p* = 0.033) Centre regions of interest. Finally, amplitude of the FRN was greater when the partner played correct notes than when the partner played wrong note errors in the Middle Centre ROI only (*t*(25) = 2.53, *p* = 0.018; see Figure SM14).

In the Posterior regions of interest, there were no main effects, but a significant interaction between Extra/Wrong/Correct and Lateralisation (*F*(2.08, 51.87) = 4.30, *p* = 0.018; see Table SM27). Amplitude of the FRN was greater when the partner played extra note errors than when the partner played wrong note errors in the Left (*t*(25) = 2.33, *p* = 0.028) and Middle (*t*(25) = 3.16, *p* = 0.004) Posterior regions of interest. Amplitude of the FRN was also greater when the partner played extra note errors than when the partner played correct notes in the Left (*t*(25) = 3.65, *p* = 0.001) and Middle (*t*(25) = 2.36, *p* = 0.026) Posterior regions of interest. There was no significant difference between correct notes and wrong note errors in any of the Posterior ROIs (all *p*-values > 0.35; see Figure SM14).

**Behavioural Results – Tables**

***Inter-keystroke Interval (IKI)***

Table SM28
*ANOVA values for behavioural analysis of self-produced IKIs.*

| Effect | *df* | *F* | *p*-value | *η_p_^2^* |
| --- | --- | --- | --- | --- |
| **E/W/C** | **(1.41, 36.61)** | **70.90** | **< 0.001** | **0.732** |
| Agency | (1, 35) | 1.16 | 0.291 | 0.043 |
| **Position** | **(1.80, 46.68)** | **56.09** | **< 0.001** | **0.683** |
| E/W/C x Agency | (1.17, 30.50) | 3.39 | 0.07 | 0.115 |
| **E/W/C x Position** | **(1.90, 49.27)** | **62.03** | **< 0.001** | **0.705** |
| Agency x Position | (1.61, 41.79) | 1.29 | 0.282 | 0.047 |
| E/W/C x Agency x Position | (1.86, 48.32) | 1.84 | 0.171 | 0.066 |

*Note:* Bold values indicate significant effects (*p* < 0.05). E/W/C = Extra note error/Wrong note error/Correct note.

Table SM29
*ANOVA values for behavioural analysis of the effect of Extra note error/Wrong note error/Correct note on self-produced IKIs at each interval position. The error keystroke terminates IKI3 and initiates IKI4.*

| Effect at each position | *df* | *F* | *p*-value | *η_p_^2^* |
| --- | --- | --- | --- | --- |
| **IKI1** | **(1.96, 50.87)** | **4.12** | **0.023** | **0.137** |
| **IKI2** | **(1.36, 35.29)** | **44.16** | **< 0.001** | **0.629** |
| **IKI3** | **(1.60, 41.49)** | **592.20** | **< 0.001** | **0.958** |
| **IKI4** | **(1.48, 38.40)** | **104.71** | **< 0.001** | **0.801** |
| IKI5 | (1.39, 36.05) | 2.30 | 0.13 | 0.081 |
| IKI6 | (1.59, 41.40) | 2.88 | 0.079 | 0.1 |
| IKI7 | (1.12, 29.13) | 0.77 | 0.403 | 0.029 |

*Note:* Bold values indicate significant effects (*p* < 0.05).

Table SM30
*ANOVA values for behavioural analysis of IKI during self performance of error and correct sequences by partner.*

| Effect | *df* | *F* | *p*-value | *η_p_^2^* |
| --- | --- | --- | --- | --- |
| E/C | (1, 35) | 1.36 | 0.252 | 0.037 |
| Agency | (1, 35) | 1.26 | 0.27 | 0.035 |
| Position | (2.86, 99.96) | 0.86 | 0.46 | 0.024 |
| E/C x Agency | (1, 35) | 3.93 | 0.055 | 0.101 |
| E/C x Position | (2.84, 99.50) | 0.46 | 0.702 | 0.013 |
| Agency x Position | (2.12, 74.14) | 0.57 | 0.575 | 0.016 |
| E/C x Agency x Position | (2.11, 73.91) | 0.51 | 0.612 | 0.014 |

*Note:* E/C = Error/Correct.

**EEG Results – Tables**

Below are the tables showing statistical values for the analyses carried out on the EEG data. ERP components analysed here are the pre-ERN/ERN, the Pe, and the FRN.

***Error-Related Negativity (pre-ERN/ERN)***

Table SM31
*ANOVA values for EEG analysis of pre-ERN during a time window of 80 to 25 ms pre-error onset.*

| Effect | *df* | *F* | *p*-value | *η_p_^2^* |
| --- | --- | --- | --- | --- |
| **E/W/C** | **(1.57, 40.87)** | **4.48** | **0.025** | **0.147** |
| Agency | (1, 26) | 0.00 | 0.966 | 0 |
| L/R | (1.49, 38.72) | 2.03 | 0.156 | 0.072 |
| A/P | (1.22, 31.83) | 2.26 | 0.138 | 0.08 |
| E/W/C x Agency | (1.26, 32.68) | 0.25 | 0.675 | 0.009 |
| **E/W/C x L/R** | **(2.87, 74.62)** | **2.90** | **0.043** | **0.1** |
| Agency x L/R | (1.88, 48.96) | 1.01 | 0.368 | 0.037 |
| E/W/C x Agency x L/R | (3.34, 86.93) | 0.57 | 0.652 | 0.022 |
| **E/W/C x A/P** | **(2.17, 56.33)** | **7.12** | **0.001** | **0.215** |
| Agency x A/P | (1.42, 36.89) | 0.25 | 0.707 | 0.009 |
| E/W/C x Agency x A/P | (1.55, 40.34) | 0.12 | 0.832 | 0.005 |
| L/R x A/P | (2.65, 68.76) | 1.49 | 0.228 | 0.054 |
| E/W/C x L/R x A/P | (3.21, 83.53) | 1.04 | 0.382 | 0.038 |
| Agency x L/R x A/P | (3.33, 86.65) | 0.35 | 0.812 | 0.013 |
| E/W/C x Agency x L/R x A/P | (3.54, 91.96) | 0.79 | 0.519 | 0.03 |

*Note:* Bold values indicate significant effects (*p* < 0.05). E/W/C = Extra note error/Wrong note error/Correct note; L/R = Left/Middle/Right; A/P = Anterior/Centre/Posterior.

Table SM32
*Statistical values for ANOVAs and follow-up analyses of pre-ERN during performance of error and correct keystrokes at a time window of 80 to 25 ms pre-error onset with factors of Extra note error/Wrong note error/Correct x Lateralization x Anterior/Posterior.*

| Effect (ANOVA) | *df* | *F* | *p*-value | *η_p_^2^* |
| --- | --- | --- | --- | --- |
| **E/W/C** | **(1.60, 41.70)** | **4.42** | **0.025** | **0.145** |
| L/R | (1.52, 39.42) | 1.49 | 0.237 | 0.054 |
| A/P | (1.19, 31.00) | 1.57 | 0.223 | 0.057 |
| E/W/C x L/R | (2.84, 73.87) | 1.98 | 0.127 | 0.071 |
| **E/W/C x A/P** | **(2.09, 54.30)** | **7.08** | **0.002** | **0.214** |
| L/R x A/P | (2.64, 68.73) | 1.45 | 0.24 | 0.053 |
| E/W/C x L/R x A/P | (3.26, 84.81) | 0.88 | 0.465 | 0.033 |
| Effect (ANOVAs) | *df* | *F* | *p*-value | *η_p_^2^* |
| Anterior: E/W/C | (1.62, 42.13) | 1.61 | 0.214 | 0.058 |
| **Centre: E/W/C** | **(1.58, 40.98)** | **3.66** | **0.044** | **0.123** |
| **Posterior: E/W/C** | **(1.63, 42.37)** | **8.79** | **0.001** | **0.253** |

*Note:* Bold values indicate significant effects (*p* < 0.05 for ANOVAs; Bonferroni corrected to *p* < 0.0167 for *t*-tests). E/W/C = Extra note error/Wrong note error/Correct; L/R = Left/Middle/Right; A/P = Anterior/Centre/Posterior.

Table SM33
*Follow-up tests of pre-ERN during performance of error keystrokes at a time window of 80 to 25 ms pre-error onset with factors of Extra note error/Wrong note error x Agency in ROIs where a difference was found between Extra note error or Wrong note error and Correct.*

| Centre Effects | *df* | *F* | *p-value* | *ηp2* |
| --- | --- | --- | --- | --- |
| **E/W** | **(1, 26)** | **5.33** | **0.029** | **0.170** |
| Agency | (1, 26) | 0.002 | 0.962 | 0 |
| E/W x Agency | (1, 26) | 0.42 | 0.522 | 0.016 |
| Posterior Effects | *df* | *F* | *p-value* | *ηp2* |
| **E/W** | **(1, 26)** | **122.47** | **0.001** | **0.331** |
| Agency | (1, 26) | 0.06 | 0.817 | 0.002 |
| E/W x Agency | (1, 26) | 0.18 | 0.674 | 0.007 |

*Note:* Bold values indicate significant effects (*p* < 0.05). E/W = Extra note error/Wrong note error.

Table SM34
*ANOVA values for analysis of ERN during a time window of 30 to 90 ms.*

| Effect | *df* | *F* | *p*-value | *η_p_^2^* |
| --- | --- | --- | --- | --- |
| E/W/C | (1.36, 35.35) | 0.34 | 0.633 | 0.013 |
| Agency | (1, 26) | 0.60 | 0.447 | 0.022 |
| L/R | (1.65, 42.87) | 0.10 | 0.869 | 0.004 |
| **A/P** | **(1.22, 31.63)** | **4.22** | **0.041** | **0.14** |
| E/W/C x Agency | (1.35, 35.01) | 0.55 | 0.513 | 0.021 |
| E/W/C x L/R | (2.44, 63.48) | 2.49 | 0.08 | 0.087 |
| Agency x L/R | (2, 51.87) | 0.11 | 0.895 | 0.004 |
| E/W/C x Agency x L/R | (2.66, 69.06) | 0.68 | 0.553 | 0.025 |
| **E/W/C x A/P** | **(1.70, 44.15)** | **4.69** | **0.019** | **0.153** |
| Agency x A/P | (1.19, 30.95) | 0.23 | 0.676 | 0.009 |
| E/W/C x Agency x A/P | (1.87, 48.53) | 0.21 | 0.796 | 0.008 |
| L/R x A/P | (2.72, 70.75) | 1.72 | 0.176 | 0.062 |
| **E/W/C x L/R x A/P** | **(3.67, 95.47)** | **2.90** | **0.03** | **0.1** |
| Agency x L/R x A/P | (2.71, 70.52) | 0.33 | 0.787 | 0.012 |
| E/W/C x Agency x L/R x A/P | (3.79, 98.41) | 1.14 | 0.342 | 0.042 |

*Note:* Bold values indicate significant effects (*p* < 0.05). E/W/C = Extra note error/Wrong note error/Correct; L/R = Left/Middle/Right; A/P = Anterior/Centre/Posterior.

Table SM35
*Statistical values for ANOVAs and follow-up analyses of ERN during performance of error and correct keystrokes at a time window of 30 to 90 ms post-error onset with factors of Extra note error/Wrong note error/Correct x Lateralization x Anterior/Posterior.*

| Effect (ANOVA) | *df* | *F* | *p*-value | *η_p_^2^* |
| --- | --- | --- | --- | --- |
| E/W/C | (1.35, 35.14) | 0.28 | 0.67 | 0.011 |
| L/R | (1.63, 42.38) | 0.05 | 0.927 | 0.002 |
| **A/P** | **(1.26, 32.64)** | **3.96** | **0.047** | **0.132** |
| E/W/C x L/R | (2.5, 64.92) | 2.3 | 0.097 | 0.081 |
| **E/W/C x A/P** | **(1.69, 43.84)** | **4.39** | **0.024** | **0.144** |
| L/R x A/P | (2.78, 72.18) | 2.25 | 0.094 | 0.08 |
| **E/W/C x L/R x A/P** | **(3.72, 96.71)** | **3.04** | **0.024** | **0.105** |
| Effect (Anterior ANOVAs) | *df* | *F* | *p*-value | *η_p_^2^* |
| Left: E/W/C | (1.58, 41.11) | 0.99 | 0.36 | 0.037 |
| **Middle: E/W/C** | **(1.75, 45.57)** | **5.64** | **0.009** | **0.178** |
| Right: E/W/C | (1.75, 45.49) | 0.61 | 0.529 | 0.023 |
| Effect (Centre ANOVAs) | *df* | *F* | *p*-value | *η_p_^2^* |
| Left: E/W/C | (1.34, 34.82) | 0.66 | 0.467 | 0.025 |
| Middle: E/W/C | (1.35, 35.08) | 1.26 | 0.284 | 0.046 |
| Right: E/W/C | (1.49, 38.80) | 0.61 | 0.504 | 0.023 |
| Effect (Posterior ANOVAs) | *df* | *F* | *p*-value | *η_p_^2^* |
| Left: E/W/C | (1.31, 33.99) | 0.54 | 0.515 | 0.02 |
| Middle: E/W/C | (1.28, 33.23) | 0.21 | 0.712 | 0.008 |
| Right: E/W/C | (1.32, 34.30) | 0.94 | 0.365 | 0.035 |

*Note:* Bold values indicate significant effects (*p* < 0.05 for ANOVAs; Bonferroni corrected to *p* < 0.0167 for *t*-tests). E/W/C = Extra note error/Wrong note error/Correct; L/R = Left/Middle/Right; A/P = Anterior/Centre/Posterior.

Table SM36
*Follow-up tests of ERN during performance of error keystrokes at a time window of 30 to 90 ms post-error onset with factors of Extra note error/Wrong note error x Agency in ROIs where a difference was found between Extra note error or Wrong note error and Correct.*

| Middle Anterior Effects | *df* | *F* | *p*-value | *η_p_^2^* |
| --- | --- | --- | --- | --- |
| **E/W** | **(1, 26)** | **8.79** | **0.006** | **0.253** |
| Agency | (1, 26) | 0.5 | 0.488 | 0.019 |
| E/W x Agency | (1, 26) | 0.1 | 0.749 | 0.004 |

*Note:* Bold values indicate significant effects (*p* < 0.05). E/W = Extra note error/Wrong note error.

***Error Positivity (Pe)***

Table SM37
*ANOVA values for EEG analysis of Pe during a time window of 120 to 230 ms.*

| Effect | *df* | *F* | *p*-value | *η_p_^2^* |
| --- | --- | --- | --- | --- |
| E/W/C | (1.30, 33.80) | 2.77 | 0.096 | 0.096 |
| Agency | (1, 26) | 0.02 | 0.902 | 0.001 |
| **L/R** | **(1.93, 50.18)** | **9.37** | **< 0.001** | **0.265** |
| A/P | (1.27, 33.00) | 3.45 | 0.063 | 0.117 |
| E/W/C x Agency | (1.56, 40.43) | 0.53 | 0.55 | 0.02 |
| E/W/C x L/R | (2.47, 64.20) | 2.40 | 0.088 | 0.084 |
| Agency x L/R | (1.57, 40.87) | 0.45 | 0.595 | 0.017 |
| E/W/C x Agency x L/R | (1.97, 51.22) | 0.66 | 0.52 | 0.025 |
| **E/W/C x A/P** | **(1.53, 39.73)** | **5.40** | **0.014** | **0.172** |
| Agency x A/P | (1.10, 28.54) | 3.04 | 0.089 | 0.105 |
| E/W/C x Agency x A/P | (2.04, 53.10) | 3.02 | 0.056 | 0.104 |
| **L/R x A/P** | **(2.62, 68.19)** | **6.14** | **0.002** | **0.191** |
| E/W/C x L/R x A/P | (4.78, 124.14) | 1.44 | 0.216 | 0.053 |
| Agency x L/R x A/P | (2.89, 75.21) | 0.10 | 0.958 | 0.004 |
| E/W/C x Agency x L/R x A/P | (4.13, 107.49) | 0.64 | 0.64 | 0.024 |

*Note:* Bold values indicate significant effects (*p* < 0.05). E/W/C = Extra note error/Wrong note error/Correct note; L/R = Left/Middle/Right; A/P = Anterior/Centre/Posterior.

Table SM38
*Statistical values for ANOVAs and follow-up analyses of Pe during performance of error and correct keystrokes at a time window of 120 to 230 ms post-error onset with factors of Extra note error/Wrong note error/Correct x Lateralization x Anterior/Posterior.*

| Effect (ANOVA) | *df* | *F* | *p*-value | *η_p_^2^* |
| --- | --- | --- | --- | --- |
| E/W/C | (1.35, 35) | 2.64 | 0.103 | 0.092 |
| **L/R** | **(1.96, 50.88)** | **10.4** | **< 0.001** | **0.286** |
| **A/P** | **(1.25, 32.51)** | **4.10** | **0.043** | **0.136** |
| E/W/C x L/R | (2.6, 67.7) | 2.80 | 0.054 | 0.097 |
| **E/W/C x A/P** | **(1.5, 39.04)** | **6.59** | **0.007** | **0.202** |
| **L/R x A/P** | **(2.42, 62.89)** | **5.88** | **0.003** | **0.185** |
| E/W/C x L/R x A/P | (4.51, 117.25) | 1.56 | 0.184 | 0.057 |
| Effect (ANOVAs) | *df* | *F* | *p*-value | *η_p_^2^* |
| **Anterior: E/W/C** | **(1.44, 37.51)** | **3.97** | **0.04** | **0.132** |
| Centre: E/W/C | (1.32, 34.2) | 3.57 | 0.057 | 0.121 |
| Posterior: E/W/C | (1.3, 33.9) | 1.23 | 0.288 | 0.045 |

*Note:* Bold values indicate significant effects (*p* < 0.05). E/W/C = Extra note error/Wrong note error/Correct; L/R = Left/Middle/Right; A/P = Anterior/Centre/Posterior.

Table SM39
*Follow-up tests of Pe during performance of error keystrokes at a time window of 120 to 230 ms post-error onset with factors of Extra note error/Wrong note error x Agency in ROIs where a difference was found between Extra note error or Wrong note error and Correct.*

| Anterior Effects | *df* | *F* | *p*-value | *η_p_^2^* |
| --- | --- | --- | --- | --- |
| E/W | (1, 26) | 2.80 | 0.106 | 0.097 |
| Agency | (1, 26) | 0.25 | 0.623 | 0.009 |
| E/W x Agency | (1, 26) | 1.07 | 0.31 | 0.04 |

*Note:* E/W = Extra note error/Wrong note error.

***Feedback-related Negativity (FRN)***

Table SM40
*ANOVA values for EEG analysis of FRN during a time window of 250 to 340 ms.*

| Effect | *df* | *F* | *p*-value | *η_p_^2^* |
| --- | --- | --- | --- | --- |
| **E/W/C** | **(1.80, 45.09)** | **6.72** | **0.004** | **0.212** |
| Agency | (1, 26) | 0.14 | 0.713 | 0.006 |
| L/R | (1.68, 41.88) | 1.07 | 0.342 | 0.41 |
| **A/P** | **(1.16, 28.91)** | **6.53** | **0.013** | **0.207** |
| E/W/C x Agency | (1.47, 36.68) | 0.04 | 0.915 | 0.002 |
| **E/W/C x L/R** | **(2.46, 61.51)** | **4.47** | **0.01** | **0.152** |
| Agency x L/R | (1.94, 48.53) | 0.74 | 0.48 | 0.029 |
| E/W/C x Agency x L/R | (2.58, 64.61) | 2.06 | 0.123 | 0.076 |
| E/W/C x A/P | (1.48, 36.91) | 3.41 | 0.057 | 0.12 |
| Agency x A/P | (1.08, 26.97) | 0.67 | 0.43 | 0.026 |
| E/W/C x Agency x A/P | (1.60, 39.96) | 1.47 | 0.241 | 0.056 |
| L/R x A/P | (3.32, 82.89) | 0.71 | 0.561 | 0.028 |
| E/W/C x L/R x A/P | (3.84, 96.01) | 1.05 | 0.386 | 0.04 |
| Agency x L/R x A/P | (2.27, 56.68) | 1.27 | 0.291 | 0.048 |
| E/W/C x Agency x L/R x A/P | (4.20, 105.03) | 0.83 | 0.515 | 0.032 |

*Note:* Bold values indicate significant effects (*p* < 0.05). E/W/C = Extra note error/Wrong note error/Correct note; L/R = Left/Middle/Right; A/P = Anterior/Centre/Posterior.

Table SM41
*Statistical values for ANOVAs and follow-up analyses of FRN during performance of error and correct keystrokes at a time window of 250 to 340 ms post-error onset with factors of Extra note error/Wrong note error/Correct x Lateralization x Anterior/Posterior.*

| Effect (ANOVA) | *df* | *F* | *p*-value | *η_p_^2^* |
| --- | --- | --- | --- | --- |
| **E/W/C** | **(1.8, 45.09)** | **6.72** | **0.004** | **0.212** |
| L/R | (1.68, 41.88) | 1.07 | 0.342 | 0.041 |
| **A/P** | **(1.16, 28.91)** | **6.53** | **0.013** | **0.207** |
| **E/W/C x L/R** | **(2.46, 61.51)** | **4.47** | **0.01** | **0.152** |
| E/W/C x A/P | (1.48, 36.91) | 3.41 | 0.057 | 0.12 |
| L/R x A/P | (3.32, 82.89) | 0.71 | 0.561 | 0.028 |
| E/W/C x L/R x A/P | (3.84, 96.01) | 1.05 | 0.386 | 0.04 |
| Effect (ANOVAs) | *df* | *F* | *p*-value | *η_p_^2^* |
| **Left: E/W/C** | **(1.77, 44.29)** | **6.35** | **0.005** | **0.203** |
| **Middle: E/W/C** | **(1.68, 41.95)** | **9.19** | **0.001** | **0.269** |
| Right: E/W/C | (1.71, 42.61) | 2.60 | 0.094 | 0.094 |

*Note:* Bold values indicate significant effects (*p* < 0.05). E/W/C = Extra note error/Wrong note error/Correct; L/R = Left/Middle/Right; A/P = Anterior/Centre/Posterior.

Table SM42
*Follow-up tests of FRN during performance of error keystrokes at a time window of 250 to 340 ms post-error onset with factors of Extra note error/Wrong note error x Agency in ROIs where a difference was found between Extra note error or Wrong note error and Correct for Left ROIs.*

| Left Effects | *df* | *F* | *p*-value | *η_p_^2^* |
| --- | --- | --- | --- | --- |
| **E/W** | **(1, 25)** | **9.31** | **0.005** | **0.271** |
| Agency | (1, 25) | 0.87 | 0.359 | 0.034 |
| E/W x Agency | (1, 25) | 0.05 | 0.818 | 0.002 |
| Middle Effects | *df* | *F* | *p*-value | *η_p_^2^* |
| **E/W** | **(1, 25)** | **15.33** | **0.001** | **0.38** |
| Agency | (1, 25) | 0.11 | 0.749 | 0.004 |
| E/W x Agency | (1, 25) | 0.01 | 0.906 | 0.001 |

*Note:* Bold values indicate significant effects (*p* < 0.05). E/W = Extra note error/Wrong note error.

**Figures – Complete Set of Stimuli (Upper Part)**


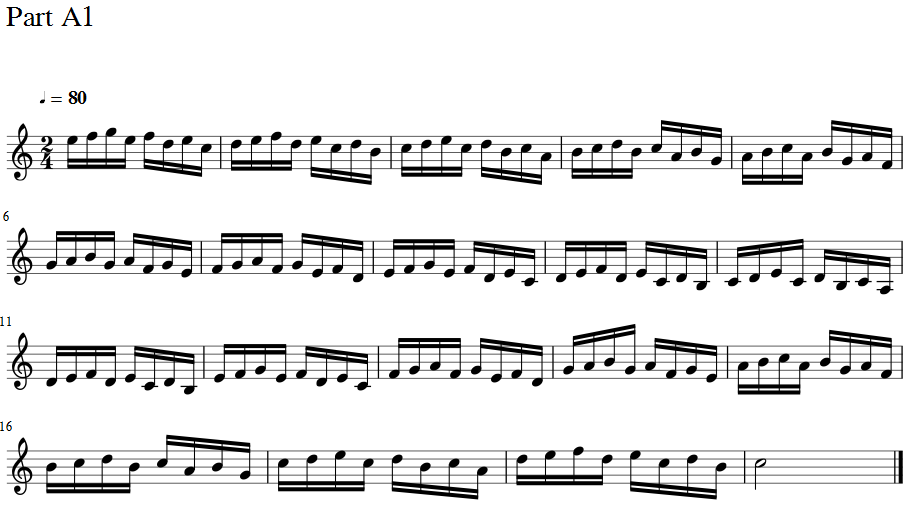


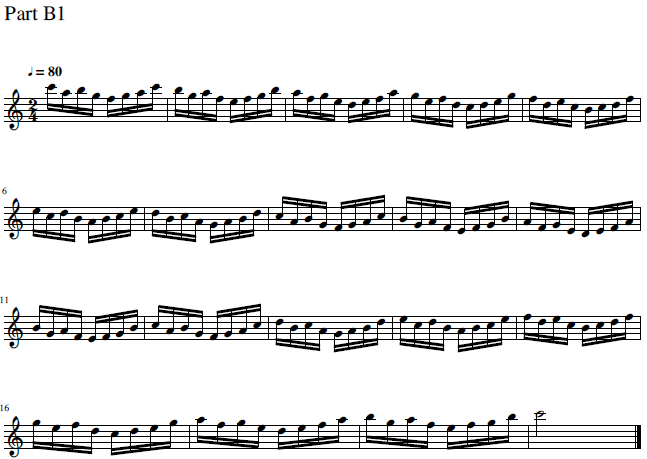


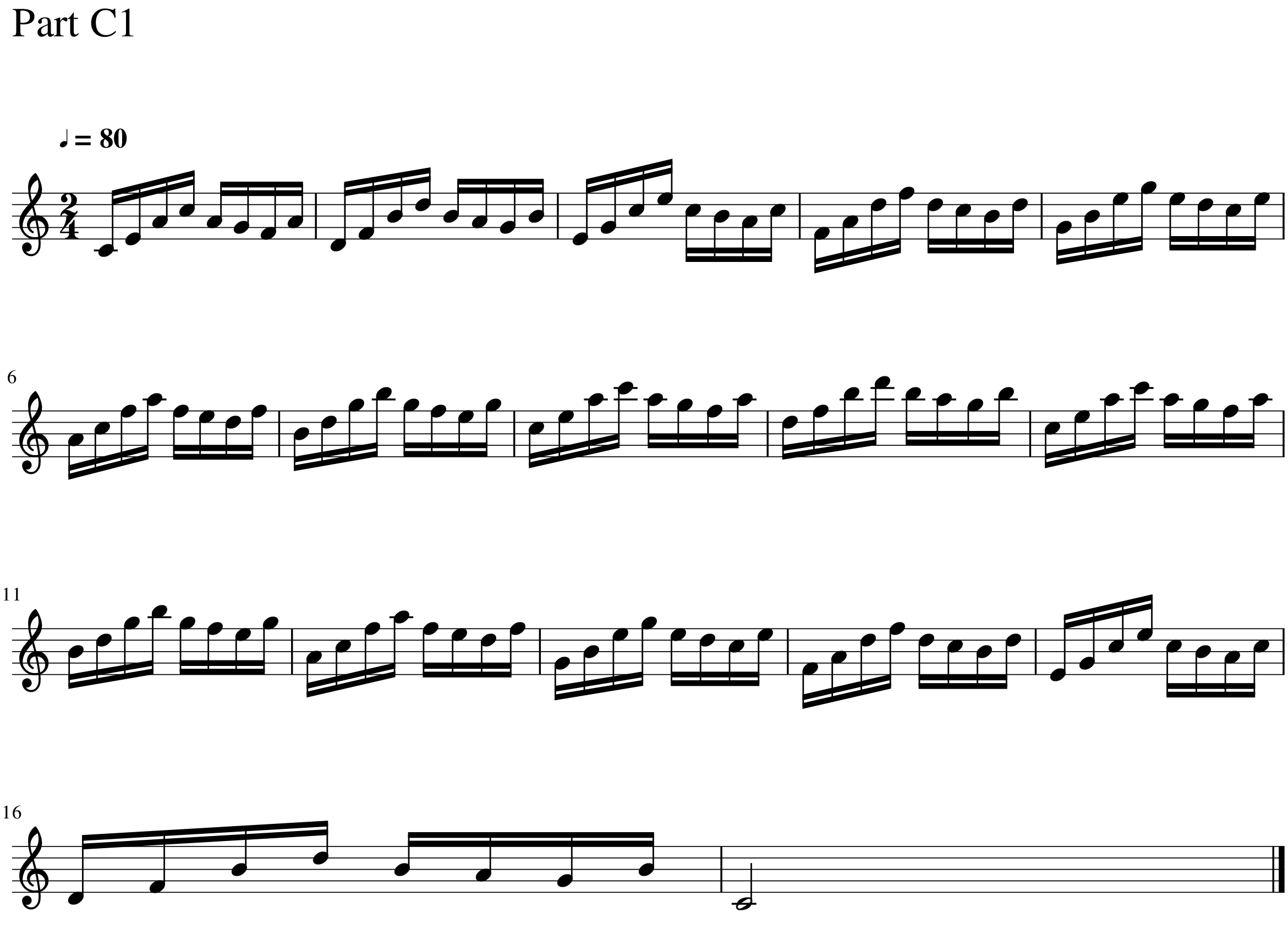


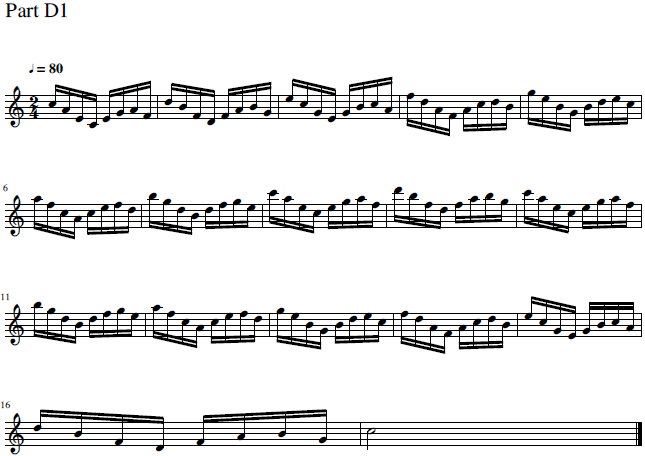


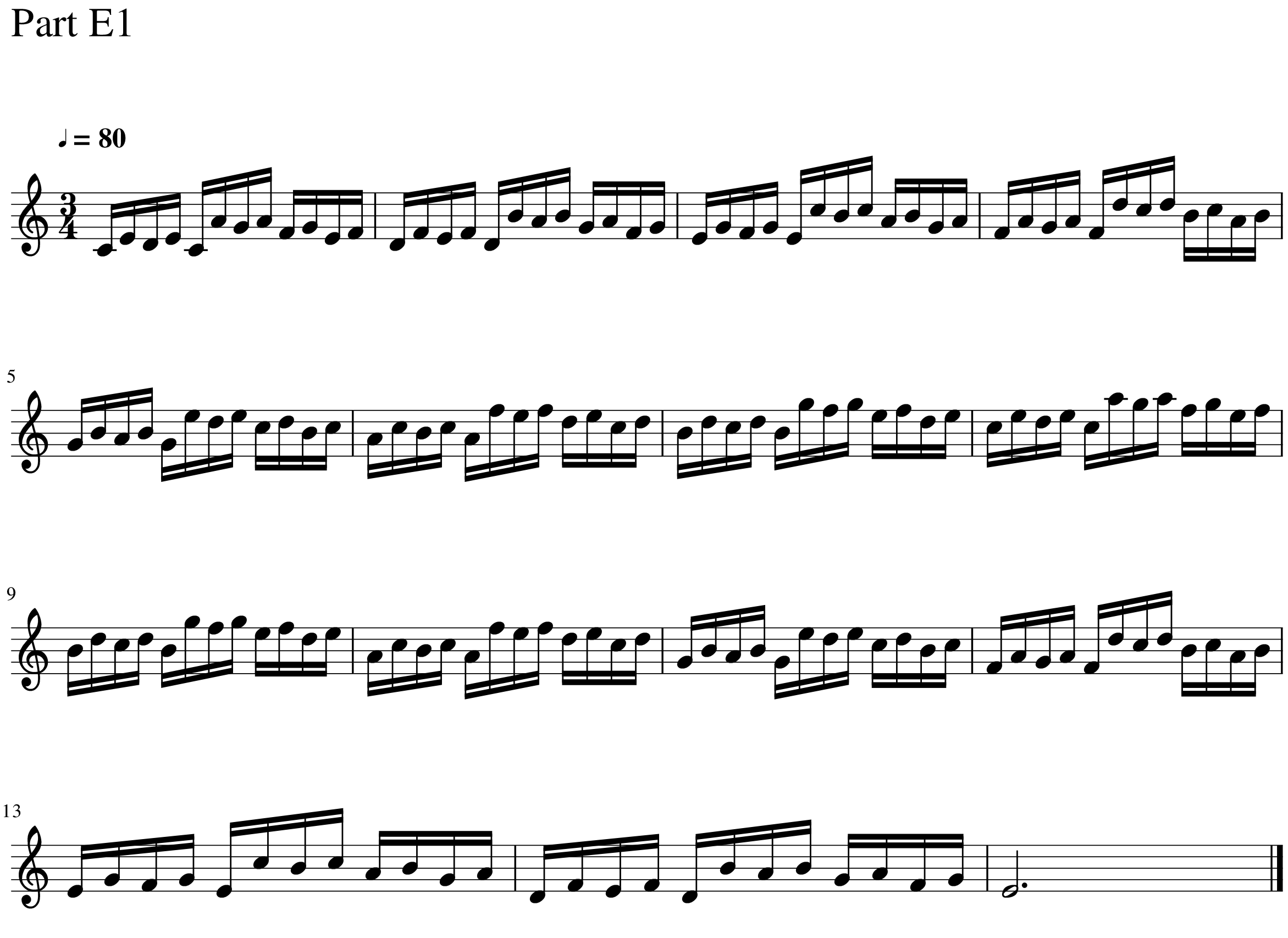


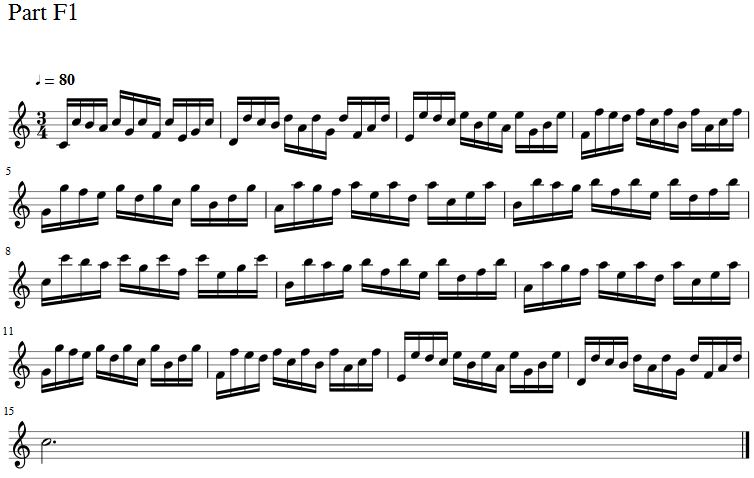


Figure SM1. The six unique piano pieces, labelled A1 – F1. A second set (A2 – F2) contained the same pieces but one octave lower in pitch.

**References**

Baker, S. L., Heinrichs, N., Kim, H. J., & Hofmann, S. G. (2002). The Liebowitz social anxiety scale as a self-report instrument: A preliminary psychometric analysis. *Behaviour Research and Therapy, 40*(6), 701-715. doi: 10.1016/S0005-7967(01)00060-2

Danielmeier, C., & Ullsperger, M. (2011). Post-error adjustments. *Frontiers in Psychology, 2*, 233. doi: 10.3389/fpsyg.2011.00233

Davis, M. (1980). A multidimensional approach to individual differences in empathy. *JSAS Catalog of Selected Documents in Psychology, 10*(85).

Fairhurst, M. T., Janata, P., & Keller, P. E. (2014). Leading the follower: An fMRI investigation of dynamic cooperativity and leader–follower strategies in synchronization with an adaptive virtual partner. *Neuroimage, 84*, 688-697. doi: 10.1016/j.neuroimage.2013.09.027

Gehring, W. J., Liu, Y., Orr, J. M., & Carp, J. (2012). The error-related negativity (ERN/Ne). In S. J. Luck & E. S. Kappenman (Eds.), *Oxford handbook of event-related potential components* (pp. 231-291). New York: Oxford University Press, Inc.

Hanon, C.-L. (1923). The Virtuoso Pianist in Sixty Exercises for the Piano. New York, NY: G. Schirmer, Inc.

Judge, T. A., Erez, A., Bono, J. E., & Thoresen, C. J. (2003). The core self‐evaluations scale: Development of a measure. *Personnel Psychology, 56*(2), 303-331. doi: 10.1111/j.1744-6570.2003.tb00152.x

Levenson, H. (1973). Multidimensional locus of control in psychiatric patients. *Journal of Consulting and Clinical Psychology, 41*(3), 397-404. doi: 10.1037/h0035357

Luck, G., Saarikallio, S., Burger, B., Thompson, M. R., & Toiviainen, P. (2010). Effects of the Big Five and musical genre on music-induced movement. *Journal of Research in Personality, 44*(6), 714-720. doi: 10.1016/j.jrp.2010.10.001

Novembre, G., Ticini, L. F., Schütz-Bosbach, S., & Keller, P. E. (2012). Distinguishing self and other in joint action. Evidence from a musical paradigm. *Cerebral Cortex, 22*(12), 2894-2903. doi: 10.1093/cercor/bhr364

Oostenveld, R., Fries, P., Maris, E., & Schoffelen, J.-M. (2011). FieldTrip: Open source software for advanced analysis of MEG, EEG, and invasive electrophysiological data. *Computational Intelligence and Neuroscience, 2011*, 9. doi: 10.1155/2011/156869

Overbeek, T. J. M., Nieuwenhuis, S., & Ridderinkhof, K. R. (2005). Dissociable components of error processing: On the functional significance of the Pe vis-à-vis the ERN/Ne. *Journal of Psychophysiology, 19*(4), 319-329. doi: 10.1027/0269-8803.19.4.319

Rammstedt, B., & John, O. P. (2007). Measuring personality in one minute or less: A 10-item short version of the Big Five Inventory in English and German. *Journal of Research in Personality, 41*(1), 203-212. doi: 10.1016/j.jrp.2006.02.001

Ruiz, M. H., Jabusch, H. C., & Altenmüller, E. (2009). Detecting wrong notes in advance: neuronal correlates of error monitoring in pianists. *Cerebral Cortex, 19*(11), 2625-2639. doi: 10.1093/cercor/bhp021

Sammler, D., Novembre, G., Koelsch, S., & Keller, P. E. (2013). Syntax in a pianist's hand: ERP signatures of “embodied” syntax processing in music. *Cortex, 49*(5), 1325-1339. doi: 10.1016/j.cortex.2012.06.007

Varlet, M., Marin, L., Capdevielle, D., Del-Monte, J., Schmidt, R. C., Salesse, R. N., . . . Raffard, S. (2014). Difficulty leading interpersonal coordination: Towards an embodied signature of social anxiety disorder. *Frontiers in Behavioral Neuroscience, 8*(29), 9. doi: 10.3389/fnbeh.2014.00029
